# Perioperative inappropriate red blood cell transfusions significantly increase total costs in elective surgical patients, representing an important economic burden for hospitals

**DOI:** 10.3389/fmed.2022.956128

**Published:** 2022-08-30

**Authors:** Andrea Saporito, Davide La Regina, Axel Hofmann, Lorenzo Ruinelli, Alessandro Merler, Francesco Mongelli, Kevin M. Trentino, Paolo Ferrari

**Affiliations:** ^1^Faculty of Biomedical Sciences, University of Italian Switzerland, Lugano, Switzerland; ^2^Division of Anesthesiology, Bellinzona e Valli Regional Hospital, Ente Ospedaliero Cantonale, Bellinzona, Switzerland; ^3^Department of Surgery, Bellinzona e Valli Regional Hospital, Ente Ospedaliero Cantonale, Bellinzona, Switzerland; ^4^Institute of Anesthesiology, University Hospital of Zurich, Zurich, Switzerland; ^5^Medical School, University of Western Australia, Perth, WA, Australia; ^6^Information and Communications Technology Unit, Bellinzona e Valli Regional Hospital, Ente Ospedaliero Cantonale, Bellinzona, Switzerland; ^7^Division of Nephrology, Lugano Regional Hospital, Ente Ospedaliero Cantonale, Lugano, Switzerland; ^8^Clinical School, University of New South Wales, Sydney, NSW, Australia

**Keywords:** costs, economy, inappropriate transfusions, red blood cell, perioperative medicine, operating room, surgery

## Abstract

**Background:**

Red blood cell (RBC) transfusions in surgical patients are associated with increased morbidity a hospital stay. However, little is known about how clinical and economic outcomes differ between appropriately and inappropriately transfused patients. We hypothesized that inappropriate RBC transfusions in elective surgical patients would significantly increase hospital cost. The aim of this study was to quantify the economic burden associated with inappropriate RBC transfusions.

**Methods:**

We retrospectively included all adult patients admitted for elective non-cardiac surgery between January 2014 and March 2020. Patients were divided into three groups (not transfused, appropriately transfused and inappropriately transfused). The primary outcome was the excess in hospital cost in patients inappropriately transfused compared to non-transfused patients. Costs were calculated using a bottom–up approach and involving cost calculation on a granular level. According to international guidelines, transfusions were considered appropriate if administered with an ASA score of 1–2 and the last hemoglobin level measured before transfusion < 70 g/L, or with an ASA score ≥ 3 and the last hemoglobin level < 80 g/L. Cases where RBC transfusions were deemed necessary regardless of the Hb levels were reviewed by the patient blood management (PBM) board and classified accordingly. Secondary outcomes included total transfusion rate, transfusion index, and length of hospital stay. Statistical analysis was carried out by multivariable regression models.

**Results:**

During the study period there were 54,922 consecutive surgical admissions, of these 1,997 received an RBC transfusion, with 1,125 considered inappropriate. The adjusted cost of each inappropriate RBC transfusions was estimated in United States dollars (USD) 9,779 (95% CI, 9,358 – 10,199; *p* < 0.001) and totaled USD 11,001,410 in our series. Inappropriately transfused patients stayed 1.6 times (95% CI, 1.5–1.6; *p* < 0.001) longer in hospital (10.6 days vs. 6.7 days) than non-transfused patients and a mean 2.35 RBC units per patient were administered.

**Conclusion:**

Inappropriate RBC transfusions in elective surgical patients seem to be common and may represent a significant economic burden. In our experience, inappropriate transfusions significantly increased hospital costs by an average of USD 9,779 compared to non-transfused patients. Through specific PBM policy, hospitals may improve cost-effectiveness of their elective surgical activity by lowering inappropriate transfusions.

## Background

The administration of allogeneic red blood cell (RBC) transfusions is an independent risk factor for morbidity and mortality (1–4). In elective surgical patients, the immunosuppressive and prothrombotic impact of RBC transfusions is associated with delayed surgical wound healing, increased nosocomial infections (5, 6) and increased perioperative cardiovascular and cerebrovascular events (7–9). These ultimately affect functional outcomes, which explains why RBC transfusions have also been recognized as an independent risk factor associated with prolonged average hospital length-of-stay (ALOS) and increased rate of readmission after discharge (3, 10, 11).

Guidelines often recommend RBC transfusion in patients with hemoglobin (Hb) thresholds < 70 g/L in clinically stable patients, and < 80 g/L in patients with cardiovascular disease or risk factors (12, 13). In addition, the evidence continues to increase about the efficacy of patient blood management (PBM) programs in reducing perioperative morbidity and mortality (14, 15), decreasing ALOS and total costs (2, 11, 14, 16). Despite this, data show persistently high variability in RBC transfusion practice worldwide (14, 15, 17, 18), with a reported high rate of inappropriate RBC transfusions (19, 20).

Inappropriate RBC transfusions still appear to be common practice (21, 22), and were the subject of several calls to action by health care organizations worldwide. Numerous medical societies and colleges have made recommendations about avoiding inappropriate transfusions (22–25). The American Association of Blood Banks’ top five recommendations encourage patients as well as their physicians to question unnecessary transfusion (23). Additionally, inappropriate RBC transfusions in surgical patients are those not matching internationally accepted criteria (12, 13), namely cases of Hb > 7 g/dL or > 8 g/dL in high-risk patients without signs of active bleeding or hemodynamic instability.

Furthermore, inappropriate RBC transfusions are also likely to have considerable impact on total treatment costs (defined as the sum of both direct and indirect costs) in surgical patients (11, 14, 16). This would be as a result of the costs of RBC transfusions, (26) and costs due to increased ALOS, unexpected readmissions, complications and a prolonged recovery after surgery (3, 27–29).

Therefore, we hypothesized that inappropriate RBC transfusions would significantly increase hospital cost. The aim of this study was to quantify the economic burden associated with inappropriate RBC transfusions for a public hospital trust, and thus estimate the potential economic impact of a PBM program aimed at eliminating inappropriate transfusions in elective surgical patients.

## Materials and methods

This was a multicenter retrospective cohort study over a 6-year period involving adult elective non-cardiac surgical admissions to the five acute care public hospitals of the Ente Ospedaliero Cantonale (EOC), Switzerland. On average, the EOC network performs 22,000 elective non-cardiac surgical procedures annually. The study was approved by the local ethics committee (BASEC-2021-00287) and written informed consent was waived as all data was fully anonymized. This manuscript adheres to STROBE guidelines.

### Participants

Adult patients undergoing elective non-cardiac surgery between January 2014 and March 2020 and with one or more hemoglobin concentration values recorded within 24 h before surgery were included. In case of more than one value, the most updated pre-transfusion Hb was used. Patients undergoing two or more surgical procedures within the same hospital stay were excluded.

### Data sources

Data were extracted from the hospital PBM Data System that was implemented in 2020 at our institution. This system collects epidemiological, clinical, and economic patient-level data from the electronic medical and administrative records, and hospital coding and billing systems. These data are updated daily and are visualized and reported via linked dashboards. The dashboard allows users to select, filter, and extract data, providing real-time trends of key indicators, such as total transfusion rate, transfusion index, rate of inappropriate transfusions, length of hospital stay, unexpected readmissions and total costs per case. The dashboard extracts and organizes data in ranges, graphs and tables, which visualize trends, and it has filters to sort data by hospital and/or department and/or type of procedure.

### Variables – outcomes

The primary outcome of interest was the excess in hospital cost in patients inappropriately transfused compared to non-transfused patients. Costs were calculated with a bottom–up approach, which involves work estimations at the lowest level of details and their aggregation to build summary totals and detailed cost assessments. This approach allows to improve substantially cost estimates, but it is resource- and time-consuming as it requires a significant amount of work. All financial values were extracted from hospital billing registries. Firstly, the whole hospitalization cost was collected, which included direct and indirect costs (30). Additionally, supplementary visits, treatments, procedures and re-admissions are calculated. Thereafter, a micr-costing technique was applied, which consisted of excluding costs of consumables not directly or indirectly related to transfusions. Costs were reported in United States dollar (USD) and included all direct and variable costs, such as the cost of RBC units, inventory management, transport and other processing fees, costs of transfusion-related complications, unexpected readmission after discharge, and proportional fix cost allocations (how indirect costs are distributed across equipment and divisions) (31). In case of death, costs were calculated as described above. Secondary outcomes were total transfusion rate, total transfusion index, total rate of inappropriate RBC transfusions, and ALOS. We defined the total transfusion rate as number of patients transfused/total number of patients and the total transfusion index as number of RBC units transfused/number of patients who received a transfusion. Exchange rate from Swiss franc to USD updated on December 26th 2021: 1.0864.

### Variables – exposures and patient blood management meeting

Patients were classified as transfused when they received one or more perioperative RBC units any time between 24 h prior and 48 h following surgery. Patients were classified according to international guidelines (6, 12, 13, 21, 25) as either appropriately or inappropriately transfused. In particular, inappropriate transfusions were defined as RBCs administered either (a) with an ASA score of 1 or 2 and the last Hb level measured before transfusion ≥ 70 g/L, or (b) with an ASA score ≥ 3 and the last Hb level before transfusion ≥ 80 g/L.

Our PBM Data System recorded all transfusion cases and marked the inappropriate ones with a “red flag.” These cases were reviewed weekly at our PBM meeting, where all physicians involved in the transfusions were asked to justify their clinical choice. Transfusions were judged either appropriate or inappropriate according to this comprehensive multidisciplinary clinical and laboratory judgment. The appropriate/inappropriate category was then corrected on the informatic system. Therefore, cases where an RBC transfusion was deemed necessary during surgery regardless of the Hb levels were always reviewed at the PBM meeting and classified accordingly. Such cases included patients with active bleeding, rapid Hb level drop or hemodynamically unstable. Secondary comparisons of interest included inappropriately transfused patients versus appropriately transfused patients, and transfused versus non-transfused patients.

### Statistical methods

For the unadjusted analysis, continuous variables were compared using the independent samples *t*-test while the chi-squared test was applied to categorical variables. For the adjusted analysis we applied linear regression models to cost outcomes and negative binomial regression to analyze length of stay. The length of stay results from the negative binomial regression models are expressed as rate ratios with 95% confidence intervals (95% CI). These ratios are interpreted as the rate of increase or decrease in length of stay for one group when compared with the reference group (not transfused). All regression models were multivariable and adjusted for age, sex, diagnosis-related group (DRG), oncology diagnosis, presence of a platelet transfusion, presence of a plasma transfusion, and ASA score. These confounders were selected *a priori* to adjust for the potential differences between transfused patients and non-transfused patients. A subgroup analysis was performed on the top 10 surgical groups. This was performed by selecting the top 10 DRGs with the greatest frequency of red cell transfusion. Statistical analyses were performed using R version 4.0.5 (The R Foundation for Statistical Computing).

## Results

Between January 2014 and March 2020 there were 54,922 consecutive adult elective surgical admissions at the five study hospitals. Patients had a mean age of 60 ± 18 years and 27,666 (50.4%) were female. Overall, 1,997 (3.6%) admissions received an RBC transfusion, with a mean 2.35 RBC units per transfused patient. The most common elective non-cardiac surgical specialty was orthopedic surgery (*n* = 15,450) followed by general surgery (*n* = 9,492) and urology (*n* = 7,509).

Patients who received RBC were older (74 vs. 59, *p* < 0.001), more likely to be female (58.0% vs. 50.1%, *p* < 0.001), had higher ASA scores (*p* < 0.001), and more likely to be an unexpected post-surgical readmission (5.9% vs. 2.1%, *p* < 0.001). The rate of appropriate and inappropriate RBC transfusion remained constant over our study period.

**FIGURE 1:**
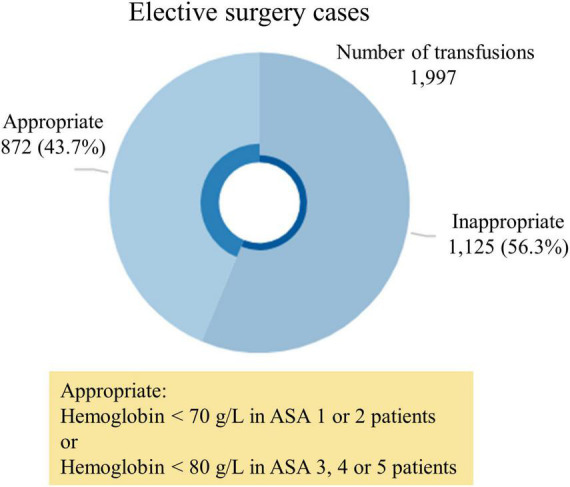
Total number of transfusions in elective patients.

**FIGURE 2:**
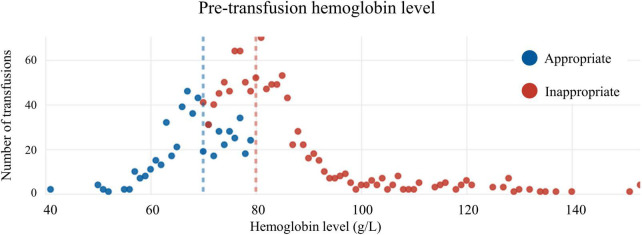
Distribution of appropriate and inappropriate transfusions according to pre-transfusion hemoglobin level.

Of patients transfused RBCs, 1,125 (56.3%) were considered to have received an inappropriate transfusion. Of patients with a Hb below 70 g/L, 31 (0.1%) did not receive a transfusion. The median pre-transfusion Hb level was 83 g/L [interquartile range (IQR) = 77 to 89 g/L] in the inappropriate transfusion group, versus 70 g/L (IQR = 66 to 76 g/L) in the appropriate transfusion group (*p* < 0.01) (Figures 1, 2). The median post-transfusion Hb level was 95 g/L (IQR = 88–104 g/L) in the inappropriate transfusion group compared to 88 g/L (IQR = 81 to 96 g/L) in the appropriate transfusion group (*p* < 0.001). Patient characteristics by RBC transfusion appropriateness are presented in Table 1. Inappropriate RBC transfusions were administered similarly according to gender, while 57% (*n* = 644) of patients aged 75 years or older were administered an inappropriate transfusion compared to 53% (*n* = 463) administered an appropriate RBC transfusion (*p* = 0.016).

**TABLE 1:** Characteristics of inpatient admissions by red blood cell transfusion appropriateness.

	Not transfused	Appropriate	Inappropriate	*P*-value
*N*	52925	872	1125	
Age, mean (*SD*)	59 (17.6)	73 (15.9)	74 (13.7)	<0.001
Sex, female (%)	26508 (50.1)	502 (57.6)	656 (58.3)	<0.001
ASA (%)				<0.001
ASA 1	9835 (18.6)	39 (4.5)	58 (5.2)	
ASA 2	27173 (51.3)	161 (18.5)	598 (53.2)	
ASA 3	15167 (28.7)	555 (63.6)	371 (33.0)	
ASA 4	746 (1.4)	115 (13.2)	96 (8.5)	
ASA 5	4 (0.0)	2 (0.2)	2 (0.2)	
Discharge type (%)				<0.001
Other	236 (0.4)	8 (0.9)	5 (0.4)	
Home care	1753 (3.3)	49 (5.6)	52 (4.6)	
Outpatient care or treatment	10962 (20.7)	101 (11.6)	118 (10.5)	
Inpatient care or treatment	1620 (3.1)	176 (20.2)	199 (17.7)	
Deceased	127 (0.2)	48 (5.5)	29 (2.6)	
Discharged home	35586 (67.2)	283 (32.5)	344 (30.6)	
Inpatient/outpatient rehab	2641 (5.0)	207 (23.7)	378 (33.6)	
Specialty (%)				< 0.001
Hepatobiliary pancreatic surgery	1022 (1.9)	7 (0.8)	8 (0.7)	
General surgery	9023 (17.0)	250 (28.7)	219 (19.5)	
Plastic and reconstructive surgery	9 (0.0)	1 (0.1)	0 (0.0)	
Thoracic surgery	781 (1.5)	16 (1.8)	20 (1.8)	
Vascular surgery	1090 (2.1)	42 (4.8)	25 (2.2)	
Visceral surgery	3101 (5.9)	47 (5.4)	47 (4.2)	
Dermatology	290 (0.5)	3 (0.3)	3 (0.3)	
Gynecology	7213 (13.6)	67 (7.7)	50 (4.4)	
Neurosurgery	3917 (7.4)	40 (4.6)	84 (7.5)	
Orthopedics	14521 (27.4)	334 (38.3)	595 (52.9)	
Otolaryngology	4578 (8.6)	7 (0.8)	3 (0.3)	
Urology	7380 (13.9)	58 (6.7)	71 (6.3)	
Red cell units, mean (*SD*)	0.00 (0.00)	2.51 (1.93)	2.23 (1.67)	<0.001
Hospital costs, USD mean (*SD*)	10,940 (9,098)	33,975 (17,485)	29,790 (15,833)	<0.001
Hospital costs, USD median [IQR]	7,961 [5,419, 13,154]	30,792 [20,090, 45,156]	25,391 [17,665, 28,526]	<0.001
Unexpected readmission (%)	1100 (2.1)	54 (6.2)	64 (5.7)	<0.001
Length of stay, median [IQR]	3 [2, 6]	15 [9, 24]	13 [9, 21]	<0.001
Intensive care unit stay (%)	3081 (5.8)	359 (41.2)	368 (32.7)	<0.001

Values are expressed as absolute number (proportion), mean with standard deviation (SD) or median with interquartile range (IQR). P-values < 0.001 refer to all comparisons.

Among transfused patients, the best rate ratios of appropriate/inappropriate transfusions were achieved by otolaryngology (ratio 2.33, 95% CI, 0.60–9.02, *p* = 0.219), vascular surgery (ratio 1.68, 95% CI, 1.03–2.74, *p* = 0.037), gynecology (ratio 1.34, 95% CI, 0.93–1.93, *p* = 0.116) and general surgery (ratio 1.14, 95% CI, 0.95–1.37, *p* = 0.147). Visceral surgery (ratio 1.00, 95% CI, 0.67–1.49, *p* = 1.000), hepatobiliary and pancreatic surgery (ratio 0.87, 95% CI, 0.32–2.40, *p* = 0.796), urology (ratio 0.82, 95% CI, 0.58–1.15, *p* = 0.251) and thoracic surgery (ratio 0.80, 95% CI, 0.42–1.53, *p* = 0.501) scored intermediate and orthopedics (ratio 0.56, 95% CI, 0.49–0.64, *p* < 0.001) and neurosurgery (ratio 0.48, 95% CI, 0.33–0.69, *p* = 0.001) scored the worst.

### Hospital costs

The full regression model equation is reported in the Supplementary Appendix 1. The mean unadjusted cost for patients transfused red cells was USD 31,617 ± 16,700, with a median of USD 27,681 (IQR = 18,300–41,776), compared with a mean of USD 10,940 ± 9,098, and a median of USD 7,961 (IQR = 5,419–13,154) for non-transfused patients. After adjusting for age, sex, ASA score, oncological diagnosis, presence of a platelet or plasma transfusion, and DRG, patients transfused red cells had higher hospital costs compared to patients not transfused (mean difference = USD 10,843, 95% CI 10,511–11,174; *p* < 0.001).

**FIGURE 3:**
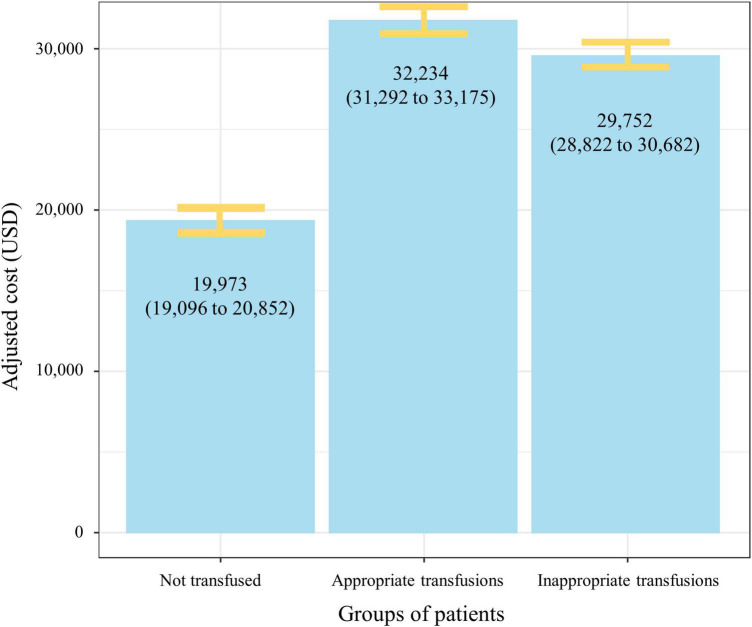
Adjusted mean hospital costs (95% CI) by red cell transfusion status. The figure demonstrates significant increase in mean hospital costs in patients transfused (appropriately and inappropriately) when compared to patients not transfused red cells. Data are adjusted for age, sex, American Society of Anesthesiologists score, oncology diagnosis, presence of a platelet transfusion, presence of a plasma transfusion, and diagnosis related group.

Hospital costs were further analyzed by transfusion status. The mean unadjusted cost for patients receiving an inappropriate RBC transfusion was USD 29,790 ± 15,834, with a median of USD 25,391 (IQR = 21,704–29,079). When excluding admissions ending in death the value was USD 29,426 ± 15,633. For patients receiving an appropriate RBC transfusion the mean unadjusted cost was USD 33,975 ± 17,485, with a median of USD 30,792 (IQR = 25,066–36,518). When excluding admissions ending in death the value was 33,439 ± 17,304. Patients not transfused had a mean unadjusted hospital cost of USD 10,940 ± 9,098, with a median of USD 7,961 (IQR = 7,735–8,186). After adjusting for potential confounders (i.e., age, sex, DRG, oncology diagnosis, presence of a platelet transfusion, presence of a plasma transfusion, and ASA score) this mean difference was significant with patients transfused inappropriately costing USD 9,779 (95% CI, 9,358–10,199; *p* < 0.001) per patient more when compared to non-transfused patients (Table 2). When excluding admissions ending in death this value was USD 9,757 (95% CI, 9,337–10,177; *p* < 0.001). The estimated additional costs of inappropriate red cell transfusions across all surgical admissions in our sample totaled USD 11,001,410 (Figure 3). The full multivariable regression model is reported in Supplementary Appendix 2.

**TABLE 2:** Regression model for incremental cost (USD) impact of appropriate and inappropriate red cell transfusion on hospital costs after adjusting for confounding factors.

Variable	Mean difference (95% CI)	*P*-value
Inappropriate RBC transfusion	9,779 (9,358, 10,199)	<0.001
Appropriate RBC transfusion	12,260 (11,783, 12,738)	<0.001
Patient age* (1-year increments)	47 (43, 51)	<0.001
Sex (female)	286 (156, 416)	<0.001
ASA class* (one class increments)	1,273 (1,181, 1,366)	<0.001
Oncology diagnosis (yes/no)	1,983 (1,657, 2,308)	<0.001
Platelet transfusion (yes/no)	1,610 (477, 2,743)	<0.001
Plasma transfusion (yes/no)	4,114 (2,804, 5,422)	<0.001
DRG family	Results too large to present, see Supplementary Appendix Table S1	

*Patient age entered into the model as a continuous variable. The mean difference in cost is interpreted as the mean difference per 1-year increase in patient age. ASA class entered into the model as a continuous variable. The mean difference in cost is interpreted as the mean difference per one-class increase in ASA score. Values are expressed in United States dollars with 95% confidence interval in parentheses.

### Average length of stay

Transfused patients had longer mean (17 vs. 5, *p* < 0.001) unadjusted ALOS when compared with non-transfused patients (median = 14 vs. 3, *p* < 0.001). ALOS was further analyzed by transfusion status. The mean unadjusted ALOS for patients receiving an inappropriate RBC transfusion was 16 days (median = 13), 18 days (median = 15) for patients receiving an RBC appropriate transfusion, and 5 (median = 3) for patients not transfused.

After adjusting for age, sex, ASA score, oncology diagnosis, presence of a platelet transfusion, presence of a plasma transfusion, and DRG, patients transfused red cells had 1.63 times (95% CI, 1.59–1.67; *p* < 0.001) longer length of stay (11.9 days vs. 6.7 days). Inappropriately transfused patients had 1.59 times (1.53–1.64; *p* < 0.001) longer length of stay than did patients not transfused (10.6 days vs. 6.7 days).

### Top 10 diagnosis-related group

The top 10 DRGs with the largest volume of admissions who received RBC transfusions are listed in Table 3. We applied regression models to each of these DRGs to estimate the difference in cost between the non-transfused and inappropriately transfused inpatients, after adjusting for confounders. The estimated additional cost associated with inappropriate RBC transfusion for the top 10 DRGs was USD 7,520 per transfused patient, representing a total of USD 4,512,298 over our study population. The greatest cost difference was in patients admitted for gastrointestinal procedures on the intestines, stomach, esophagus and duodenum (DRG G18), with the presence of an inappropriate RBC transfusion adding USD 14,616 per admission.

**TABLE 3:** Incremental costs (USD) of inappropriate red cell transfusion for top 10 transfused diagnosis-related groups (DRGs).

	Number (% Inapp. transfused)	Adjusted cost without transfusion	Adjusted cost with inappropriate transfusion	Adjusted incremental cost	Estimated cost attributable to inapp. transfusion
I08 – Other interventions on hip and femur	602 (19.3%)	22,218 (17,641, 26,796)	31,715 (27,187, 36,244)	9,497	1,101,666
I47 – Implantation or revision of a hip endoprosthesis	832 (14.3%)	19,845 (16,287, 23,403)	22,498 (18,949, 26,046)	2,652	315,603
I46 – Implantation, replacement or revision of a hip endoprosthesis	1043 (7.9%)	35,899 (27,480, 44,320)	41,040 (32,624, 49,458)	5,142	421,612
I43 – Implantation of an endoprosthesis involving the knee	1120 (7.4%)	20,983 (16,151, 25,815)	26,680 (21,703, 31,656)	5,697	472,831
G18 – Interventions on the small and large intestine or other intervention on the stomach, esophagus and duodenum	1089 (4.0%)	12,973 (2,249, 23,697)	27,589 (16,625, 38,552)	14,616	643,102
I05 – Joint replacement or revision involving the upper extremities	375 (9.9%)	27,586 (17,047, 38,126)	33,951 (23,405, 44,496)	6,365	235,493
I03 – Revision or replacement of the hip with complicating diagnosis	135 (26.7%)	23,941 (8,684, 39,197)	32,775 (17,085, 48,467)	8,836	318,086
A95 – Geriatric acute rehabilitation with OR procedure	126 (20.6%)	54,744 (37,096, 72,390)	64,090 (46,445, 81,736)	9,346	243,007
I13 – Interventions on the humerus, tibia, fibula and ankle	1472 (1.9%)	16,601 (10,156, 23,046)	23,330 (16,666, 29,933)	6,699	187,561
I09 – Spinal fusion	831 (3.5%)	21,467 (13,913, 29,022)	31,160 (23,258, 39,061)	9,692	281,076
**Total for top 10 DRGs**	**7625 (7.9%)**	**23,993** **(21,470, 26,516)**	**31,513** **(28,961, 34,066)**	**7,520**	**4,512,298**
**Total admitted episodes**	**54922 (2.1%)**	**19,973** **(19,096, 20,852)**	**29,752** **(28,822, 30,682)**	**9,779**	**11,001,410**

Mean differences and incremental costs adjusted for age, sex, ASA score, oncology diagnosis, presence of a platelet transfusion, and presence of a plasma transfusion. Values are expressed in United States dollars (USD) with 95% confidence interval in parentheses.

## Discussion

In our hospital network, inappropriate RBC transfusions were common, and added USD 9,779 per patient admission (USD 2,003,097/10,000 surgical patients), even after adjusting for a number of factors that might also explain increased cost. In our elective surgical population, this represented an estimated cost attributable to inappropriate RBC transfusions of USD 1,760,226 per annum.

Numerous studies have identified that RBC transfusions are associated with increased hospital costs, increased ALOS, increased rate of perioperative complications, and higher risk of unexpected readmission (17, 27–29). To our knowledge, however, this is the first study to specifically focus on the total costs associated with inappropriate RBC transfusions. These findings are significant as inappropriate transfusions may represent the ideal starting point for a PBM program.

In our study, we found that more than a half of patients were inappropriately transfused. Such a high rate is similar to other published data (14, 32). The reason should be sought mainly in cultural and experience-based approaches by clinicians, where previous and less restrictive guidelines may have contributed. Therefore, implementing PBM programs in clinical practice is of utmost importance to achieve more restrictive transfusion policies and reduce complications and costs. In fact, restrictive transfusion practices have been described to reduce up to 40–65% of RBC transfusions (27, 33). This outcome was achieved by education, clinical decision support, and a multidisciplinary approach. Frank et al. (34) implemented the education of clinicians, decision support tools, audits for guideline compliance, and other blood conservation measures to reduce transfusion need. Saag et al. (35) implemented a combined approach based on decision support and targeted education, which included a mandatory selection of clinical indications in every transfusion order and evidence-based information to hospital services. Finally, choosing wisely policies underlined the importance of transfusing one unit of RBC rather than two in many clinical scenarios among several other recommendations (22, 24, 25).

Our findings are consistent with other economic evaluations estimating the impact of RBC transfusion on total hospital costs. For example, after adjusting for patient differences (including DRGs) RBC transfusion is estimated to add 50–80% to total hospital costs (16, 36). In our study, transfused patients had 70% higher costs after accounting for confounders. Our study, however, further distinguishes between appropriate and inappropriate RBC transfusions and therefore focuses on the economic impact of deviance from international recommendations.

One unexpected finding was that the incremental costs associated with patients receiving inappropriate transfusion were similar to the costs associated with appropriate transfusion. This may be due to the small actual difference in pre-transfusion Hb levels between the two groups (70 g/L vs. 80 g/L). Patients receiving appropriate transfusions are those with more comorbidities and lower levels of Hb. The severity of illness, although adjusted for many variables, may account for the slightly higher cost found in patients appropriately transfused. A more comprehensive regression model or propensity score-matched analyses are expected to flatten such differences. Furthermore, one study found the transfusion of even one unit of red cells increased the risk of postoperative complications and length of stay (and presumably cost) compared with matched patients not transfused (37). This may suggest that hospital costs are higher in patients transfused regardless of the appropriateness of the transfusion.

Inappropriate RBC transfusions were also independently associated with increased ALOS when compared to non-transfused patients. This relationship may be a result of an increased incidence of complications; both transfusion-related procedural complications and transfusion-specific delayed complications due to the known effects of allogeneic blood on homeostasis, rheology and immune system function (38, 39).

Data of our study was extracted from DRG hospital billing registries. A specific DRG included all costs patients incurred for a treatment, including direct and indirect costs of hospitalization, operations, medications, procedures, etc., which were calculated for every patient. The cost calculation we performed was based on the Swiss-DRG (30). Different DRG systems share the same concept and have many similarities (31). Moreover, several articles described that DRG systems in Europe and United States are comparable in terms of cost calculation and DRG-based reimbursement. Although cost values vary among countries, economic analyses are applicable to countries adopting DRG systems (30, 31, 40).

In the population studied, we estimate a total cost of USD 11,001,410 within the 6-year study period may be attributable to inappropriate RBC transfusions. A PBM policy targeting inappropriate transfusions in the elective surgical patient population has thus a potentially significant economic impact for a hospital. Extrapolating from our results, for a facility with an average elective surgical case-load of about 10,000 patients per year and an inappropriate transfusion rate similar to ours, the potential for saving in total hospital costs can be estimated as approximately USD 1.7 million per year.

Furthermore, while our study focused on inappropriate transfusions, studies have demonstrated that over half of appropriate RBC transfusions may be avoided in elective surgery with the implementation of a preoperative clinic to screen and manage anemia and suboptimal iron stores (32). Future economic evaluations will investigate the clinical and economic implications of these interventions.

Our study has some limitations. The first one is the retrospective analysis of a prospectively collected database. With our definition of appropriate and inappropriate RBC transfusions, some cases might be inaccurate. However, we adhered to international guidelines and all inappropriately transfused cases were marked with “red flags” by our informatic system and reviewed weekly at our PBM meeting. A comprehensive multidisciplinary clinical and laboratory assessment of each case was carried out and if the RBC transfusion was deemed necessary (e.g., active bleeding, hemodynamic instability) the transfusion was classified as appropriate, thus reducing the chance of being mislabeled. Although subjectiveness in the clinical judgment of “red flag” cases might exist, it should be limited as every case was thoroughly reviewed at the multidisciplinary PBM meeting. The ASA score that was used to classify patients with pre-transfusion Hb between 70 g/L and 80 g/L may leave some inaccuracies in a very small proportion of cases. However, the ASA score is a reliable surrogate for assessing the severity of illness, especially for old and frail patients. Due to its simplicity, it is universally adopted worldwide and is important for the readability and generalizability of our results. Another limitation is related to the period chosen for inclusion in the study. We selected only patients who received a transfusion within 24 h before and 48 h after surgery. Widening this time length (e.g., entire hospitalization) would identify many transfusions not related to the surgical procedure, therefore, it would add confounders and likely result in a biased analysis. We considered the most updated pre-transfusion Hb level, therefore, differences up to 24 h in time length before receiving the transfusion may exist among patients. Another limitation is the selection of patients undergoing one surgical procedure only during the same hospital stay. Patients undergoing more than one elective surgery are generally treated within different hospitalizations and, therefore, included in the analysis. On the other hand, patients suffering from major surgical complications are expected to undergo urgent surgery and to suffer from further complications that should be due to the surgery itself rather than the RBC transfusions. Excluding patients with two or more surgeries is key to minimize biases and to focus on the economic burden of inappropriate RBC transfusions. A subgroup analysis separating preoperative vs. intra- and postoperative transfusions would be of interest. However, such data cannot be retrieved from our PBM informatic system and it should be assumed that the vast majority of patients received only intra- and postoperative transfusions. Almost all RBC transfusions in our series were cross-matched, however, emergency cases requiring RBC may have been transfused with 0 negative blood. These cases would be expected in the appropriate RBC transfusion group, but the actual data was not available and its clinical relevance remains underexplored. Another limitation is represented by the fact that we could not extract data about the incidence of RBC transfusions-related complications in the two groups; preventing us from drawing statistically supported conclusions about the possible causes of the observed data.

A further limitation exists on how costs were calculated. We collected the cost of the whole hospitalization and we excluded all costs for consumables that were not directly attributable to transfusions. The calculated cost cannot be considered the “real” cost associated to transfusions but rather a reliable surrogate that needs to include several fixed cost not collectible otherwise. The bottom-up approach, although time- and resources consuming, allowed us to improve substantially our cost estimates. Finally, we could not retrieve the length of intensive care unit stay and had to include in the analysis the incidence of intensive care unit admission only.

## Conclusion

Despite limitations, inappropriate RBC transfusions in adult elective surgical patients seem to be common in the clinical practice and may represent an economic burden. In our experience, inappropriate transfusions significantly increased hospital costs by an average of USD 9,779 compared to non-transfused patients. Through specific PBM policy, hospitals may improve cost-effectiveness of their elective surgical activity by lowering inappropriate transfusions rate.

## Data availability statement

The raw data supporting the conclusions of this article will be made available by the authors, without undue reservation.

## Ethics statement

The studies involving human participants were reviewed and approved by Comitato Etico Cantonale Ticino (BASEC-2021-00287). Written informed consent for participation was not required for this study in accordance with the national legislation and the institutional requirements.

## Author contributions

AS, DL, AH, and PF: protocol and project development. LR, AM, FM, and KT: data acquisition and interpretation. KT and FM: statistical analysis. AS, DL, FM, KT, LR, and AM: manuscript drafting. AS, PF, DL, and AH: manuscript revision and accountable for all aspects of the work. All authors: approved the final version of the manuscript.
