# Supplementary material for: Perioperative inappropriate red blood cell transfusions significantly increase total costs in elective surgical patients, representing an important economic burden for hospitals
Source: Front Med (Lausanne). 2022 Aug 30;9:956128. doi: 10.3389/fmed.2022.956128 (PMC9468475; doi:10.3389/fmed.2022.956128)
Supplement: Supplementary file 1 [file Table_1.DOCX]

**Supplemental appendix 1: Full regression model equation**

Incremental cost (USD) = 13’275.2 + (Appropriate RBC transfusion)*12’260.2 + (Inappropriate RBC transfusion)*9’778.6 + (No RBC Transfusion)*1 + (AGE, years)*47.0 + (Sex, female)*285.9 + (ASA Class)*1’273.2 + (Oncology diagnosis, yes)*1’982.8 + (Platelet transfusion, yes)*1’610.1 + (Plasma transfusion, yes)*4’113.5 + (Wound debridement for injuries with major complication or comorbidity, yes)*1 + (Wound debridement for injuries with complication or comorbidity, yes)*-5’640.50 + (Prolonged assisted ventilation, yes)*36’079.90 + (Assisted ventilation, yes)*36’593.40 + (Bone marrow transplant, yes)*16’544.50 + (Complex intensive care unit, yes)*33’416.60 + (Complex negative pressure treatment, yes)*33’801.40 + (Transplant rejection, yes)*-9’653.40 + (Radiotherapy, yes)*30’106.70 + (Acute geriatric rehabilitation, yes)*11’348.40 + (Complex palliative medicine treatment, yes)*20’563.30 + (Complex OR procedures for diseases and disorders of the nervous system, yes)*31’593.30 + (Complex craniotomy or spine surgery, yes)*19’710.70 + (Operations for para- or quadriplegia, brain disease, and interventions for cerebral palsy, muscular dystrophy, neuropathy, or operations on the spine and spinal cord surgery, yes)*395.6 + (Extracranial vessel surgery, ASD closure or cardiac pacemaker closure , yes)*-2’016.10 + (Decompression for carpal or tarsal tunnel syndrome, yes)*-15’841.60 + (Rubella with neurological complications, yes)*-6’037.90 + (Other cranial surgery, yes)*4’159.70 + (Radiotherapy for diseases and disorders of the nervous system, yes)*12’988.80 + (Peripheral and cranial nerve surgery or complex skin surgery or implant removal, yes)*-12’261.80 + (Chronic viral hepatitis, yes)*1’607.90 + (Craniotomy or major spine surgery, yes)*8’849.00 + (Neurostimulator implant, yes)*-9’313.70 + (Drug infusion pump implant, yes)*-11’848.80 + (Nervous system diseases and disorders with complex critical care, yes)*40’346.50 + (Complex neurologic treatment of acute stroke with surgery, yes)*12’638.10 + (Specific acute diseases and traumas of the spinal cord, yes)*14’534.20 + (Dementia and other chronic disorders of brain function, yes)*-4’047.40 + (Delirium, yes)*-5’387.70 + (Neoplasms of the nervous system or stupor and coma of non-traumatic origin, yes)*-16’526.10 + (Transient cerebral ischemia and occlusion of extracranial vessels, yes)*-8’820.50 + (Apoplexy, yes)*10’743.30 + (Cranial and peripheral nerve diseases, yes)*-12’008.40 + (Nervous system infection except viral meningitis, yes)*-1’016.90 + (Epileptic seizures or video-EEG or complex epilepsy treatment, yes)*-8’024.80 + (Cephalea, yes)*-12’853.90 + (Intracranial trauma, yes)*2’717.40 + (Vascular myelopathies or other diseases of the nervous system, yes)*9’775.40 + (Degenerative diseases of the nervous system or cerebral palsy, yes)*-2’781.70 + (Orbit and ocular bulb surgeries, yes)*-11’937.50 + (Eyelid surgery, yes)*-11’066.50 + (Interventions on the lacrimal apparatus, yes)*-9’203.90 + (Conjunctive surgeries or other procedures, yes)*-8’354.50 + (Malignant Tumor: Stomach Body, yes)*-3’367.70 + (Malignant cancer of the rectum, yes)*-11’656.80 + (Bimaxillary osteotomy and complex jaw surgery, yes)*5’443.30 + (Complex salivary gland operations, yes)*-8’633.90 + (Challenging surgeries on sinuses, mastoid, middle ear and other procedures on salivary glands, yes)*-9’913.30 + (Oral cavity and mouth surgery for malignant neoplasm, yes)*-12’671.30 + (Tonsillectomy for malignant neoplasm or miscellaneous ear, nose, mouth and throat, yes)*-14’187.30 + (Other ear, nose, mouth, and throat surgeries, yes)*-11’830.00 + (Tonsillectomy or adenectomy except for malignant neoplasm, yes)*-12’102.70 + (Tracheostomy or irradiation session for multidrug-resistant pathogen, yes)*15’937.30 + (Removal of osteosynthesis material from jaw and face, yes)*-15’363.50 + (Oral cavity and mouth surgeries except for malignant neoplasm, yes)*-14’334.40 + (Complex skin plastics and major head and neck surgeries, yes)*8’282.20 + (Moderately complex head and neck surgeries, radiation therapy with surgery, yes)*-11’519.90 + Single-maxillary osteotomy and complex head and neck or other surgery on the head and neck for malignant neoplasm, yes)*-4’819.80 + (Jaw surgery and other head and neck surgery except for malignant neoplasia, yes)*-13’106.00 + (Ear, nose, mouth, and throat surgeries except for malignant neoplasm malignant, without plastic surgery of the buccal floor, yes)*-13’761.60 + (Nose and sinus surgeries for malignant neoplasia, yes)*-3’474.00 + (Diseases and disorders of the ear, nose, mouth, and throat with complex intensive therapy, yes)*13’745.60 + (Complex and very complex operations on the nose, yes)*-11’860.70 + (Other nose surgeries, yes)*-13’607.90 + (Tooth extraction and repair, yes)*-16’935.60 + (Malignant neoplasms of the ear, nose, mouth and throat, yes)*-15’211.00 + (Epistaxis or otitis media or upper airway infections, yes)*-9’917.80 + (Laryngotracheitis and epiglottitis, yes)*-12’323.90 + (Traumatism and deformities of the nose, yes)*-11’826.70 + (Other diseases of the ear, nose, mouth, and throat, yes)*-15’051.80 + (Diseases of the teeth and oral cavity without extraction and repair of teeth, yes)*-13’541.60 + (Revision surgery, bilateral lobectomy, yes)*8’807.80 + (Other respiratory procedures or major bronchus surgery, yes)*-4’076.30 + (Other major chest surgery or extracorporeal circulation, yes)*4’676.90 + (Other lung resection, thoracic organ biopsy, and chest wall surgery thoracic wall, yes)*-5’484.60 + (Interventions for sleep apnea, yes)*-14’454.40 + (Radiotherapy for respiratory diseases and disorders, yes)*-13’868.00 + (Respiratory diseases and disorders with complex intensive care unit, yes)*24’102.50 + (Malnutrition, yes)*17’354.40 + (Respiratory insufficiency or pulmonary embolism, yes)*-13’840.50 + (Chronic obstructive pulmonary disease, yes)*-4’638.40 + (Severe chest trauma, yes)*-4’992.10 + (Acute bronchitis, yes)*-16’393.00 + (Respiratory tract neoplasms, yes)*-5’239.80 + (Pleural effusion, yes)*9’082.30 + (Interstitial lung disease, yes)*-5’260.60 + (Other respiratory diseases, yes)*-10’332.00 + (Tuberculosis or pneumothorax, yes)*-4’885.00 + (Other respiratory tract infections and inflammations, yes)*8’696.40 + (Control and optimization of a pre-existing artificial respiration therapy, yes)*-15’325.60 + (Challenging multi-time procedures or complex negative pressure therapy, yes)*18’372.70 + (Reconstructive vascular procedures or thoracoabdominal aneurysm with complex aortic procedure , yes)*5’609.60 + (Cardiac pacemaker implantation, yes)*-3’427.00 + (Amputation for circulatory disease of the upper extremity and toes for diabetes mellitus with complications, yes)*10.1 + (Complex or multiple vascular interventions, yes)*-11.6 + (Cardiac pacemaker replacement, yes)*-6’569.30 + (Revision of cardiac pacemaker or automatic defibrillator, yes)*-12’851.70 + (Bilateral ligation and stripping of veins with ulceration, yes)*-9’229.70 + (Other procedures for diseases of the cardiovascular system, yes)*-3’901.10 + (Amputation for circulatory diseases except upper limb and toes, yes)*14’641.20 + (Reconstructive vascular procedures , yes)*4’815.30 + (Other major reconstructive vascular procedures, yes)*591.3 + (Other cardiothoracic procedures, yes)*-1’021.80 + (Diseases and disorders of the cardiovascular system with complex critical care, yes)*25’370.70 + (Other Behavior Disorders, yes)*-716 + (Vein ligation and stripping, yes)*-12’686.80 + (Invasive cardiologic diagnostics for acute myocardial infarction, yes)*-1’591.90 + (Vegetative dysfunction of the cardiovascular system, yes)*31’273.50 + (Endovascular stent insertion in the aorta, yes)*3’050.30 + (Psychological and behavioral factors associated with disorders or diseases, yes)*-4’425.00 + (Complex or moderately complex vascular interventions, yes)*-4’658.80 + (Acute myocardial infarction without invasive cardiological diagnostics, yes)*8’358.30 + (Endocarditis, yes)*-760.9 + (Heart failure and shock, yes)*4’774.50 + (Venous Thrombosis, yes)*-6’055.30 + (Skin Ulcer on Circulatory Diseases, yes)*-2’786.00 + (Peripheral vascular diseases, yes)*13’871.80 + (Arterial Hypertension, yes)*-4’682.20 + (Moderate mental impairment, yes)*-9’200.40 + (Severe mental impairment, yes)*-12’540.20 + (Very severe mental impairment, yes)*-10’908.90 + (Other diseases of the cardiovascular system, yes)*-11’683.60 + (Complicated surgery or diagnosis on intestine, yes)*7’901.50 + (Major operations on stomach, esophagus and duodenum with highly complex surgery, yes)*16’034.60 + (Minor interventions on small intestine and colon, yes)*5’731.30 + (Appendectomy for peritonitis, yes)*-10’027.80 + (Complex reconstruction of the abdominal wall, yes)*-7’708.90 + (Hernia surgery, yes)*-12’080.00 + (Specific interventions on the hepatobiliary system, pancreas, spleen, yes)*2’336.70 + (Pyloromyotomy or anoproctoplasty and reconstruction of anus and sphincter, yes)*-10’225.10 + (Other digestive tract procedures with complex procedure, yes)*-5’152.30 + (Other bowel surgery or enterostomy, yes)*19’110.40 + (Complex rectal resection or pelvic evisceration or surgery for malignant neoplasm, yes)*12’039.00 + (Other rectal resection without specific intervention, yes)*3’718.90 + (Small bowel and colon or other surgery on the stomach, esophagus, and duodenum, yes)*-1’633.00 + (Other stomach, esophagus, and duodenum surgeries for malignant neoplasm, yes)*-2’767.50 + (Surgery for adhesions, yes)*-8’600.20 + (Appendectomy on peritonitis, yes)*-10’317.60 + (Appendectomy except on peritonitis, yes)*-11’231.00 + (Abdominal wall, umbilical and other hernia surgeries, yes)*-12’911.40 + (Inguinal and femoral hernia surgery, yes)*-12’510.50 + (Other anus surgeries, yes)*-13’243.50 + (Other radiation therapy for diseases and disorders of the digestive system, yes)*36’298.60 + (Complex multi-stage procedures for digestive diseases and disorders, yes)*4’766.10 + (Complex negative pressure therapy for digestive diseases and disorders, yes)*32’668.80 + (Multivisceral surgery for diseases and disorders of the digestive system, yes)*22’620.80 + (Complicating procedures or complex intensive care with specific surgery for diseases and disorders of the digestive system, yes)*25’526.80 + (Gastroscopy of various types for severe digestive diseases associated with complicating procedures, yes)*-7’546.70 + (Other gastroscopy for serious diseases of the digestive system, yes)*-10’720.00 + (Colonoscopy or complicating surgery, yes)*1’682.90 + (Malignant neoplasm of the digestive system, yes)*-9’882.00 + (Inflammatory bowel disease or other serious disease of the digestive system, yes)*2’639.50 + (Digestive tract obstruction, yes)*-13’196.90 + (Digestive diseases or gastrointestinal bleeding, yes)*-1’386.80 + (Other severe diseases of the digestive system, yes)*-11’579.50 + (Other digestive diseases of moderate severity, yes)*-14’153.20 + (Other mild to moderate digestive diseases or abdominal pain, yes)*-12’750.00 + (Gastrointestinal bleeding or peptic ulcer, yes)*-5’167.20 + (Pancreas and liver surgery with complex surgery, yes)*18’452.00 + (Complex gallbladder and biliary tract surgeries, yes)*13’251.50 + Laparotomy and moderately complex gallbladder and biliary tract surgery, yes)*1’436.60 + (Other hepatobiliary and pancreas procedures, yes)*-9’733.40 + (Cholecystectomy with complex diagnostic, yes)*-10’084.30 + (Laparoscopic cholecystectomy with complex diagnostic, yes)*-10’859.90 + (Pancreas and liver surgeries and portosystemic shunts, yes)*6’302.10 + (Miscellaneous hepatobiliary surgeries with extremely serious complications, yes)*1’586.70 + (Complex multi-stage procedures for hepatobiliary and pancreatic diseases and disorders, yes)*12’334.10 + (Complex therapeutic ERCP, yes)*-3’238.50 + (Liver cirrhosis and non-infectious hepatitis , yes)*-6’218.90 + (Malignant neoplasm of the hepatobiliary system and pancreas, yes)*-8’035.20 + (Diseases of the pancreas except malignant neoplasms with acute pancreatitis or liver cirrhosis, yes)*-14’428.40 + (Liver diseases except malignant neoplasms, liver cirrhosis, and noninfectious hepatitis, yes)*-7’775.80 + (Gallbladder and biliary tract diseases with extremely serious complications, yes)*-12’613.20 + (Challenging Tissue/Skin Transplantation, yes)*22’579.10 + (Hip revision or replacement with complicating diagnosis, yes)*2’299.10 + (Knee revision or replacement with complicating diagnosis, yes)*5’120.10 + (Other major joint replacement or revision or hip replacement, yes)*-4’502.70 + (Complex vertebral fusion, yes)*15’249.90 + (Amputation, yes)*8’067.60 + (Other multiple hip and femur surgeries, yes)*-3’744.30 + (Vertebral fusion, yes)*-378.1 + (Other spine surgeries, yes)*-9’039.60 + (Limb lengthening surgery, yes)*-3’439.20 + (Infection / inflammation of bones and joints, yes)*2’525.90 + (Multiple surgeries on humerus, tibia, fibula, and ankle, yes)*-5’665.30 +

(Amputation stump revision, yes)*-3’412.40 + (Cranial and facial bone surgeries, yes)*-1’791.10 + (Other shoulder or clavicle surgeries, yes)*-10’691.70 + (Facial bone surgeries, yes)*-9’638.40 + (Arthroscopy including biopsy or other bone or joint surgery, yes)*-12’460.20 + (Vertebral body fusions, yes)*5’988.50 +

(Multiple highly complex foot surgeries, yes)*-9’879.00 + (Specific hip and femur or complex elbow and forearm surgeries, yes)*-11’111.90 + (Tissue/skin transplant with complicating procedures or surgery on multiple locations, yes)*5’623.80 + (Local excision and removal of osteosynthesis material, yes)*-13’090.70 + (Diagnostic procedures on bones and joints, including biopsy, yes)*-6’245.90 + (Soft tissue surgeries for malignant neoplasms, yes)*-10’539.70 + (Other bone or connective tissue surgeries, yes)*-967.7 + (Complex shoulder surgeries, yes)*-10’785.00 + (Complex knee surgeries, yes)*-8’086.30 + (Complex multiple elbow and forearm surgeries, yes)*-7’723.80 + (Wrist and hand surgeries with complex or moderately complex multi-stage surgery, yes)*-10’351.80 + (Rehabilitation treatment for diseases and disorders of the musculoskeletal system and connective tissue, yes)*21’840.90 + (Bilateral hip or knee implantation, yes)*1’758.60 + (Infusions for Musculoskeletal Disorders, yes)*-2’893.80 + (Knee endoprosthesis implantation with prosthesis replacement, yes)*-4’006.80 + (Implant in knee endoprosthesis component replacement, yes)*-6’970.70 + (Implantation and replacement of disc prosthesis, more than one segment, yes)*-9’810.00 + (Implantation, replacement or revision of a hip prosthesis with complex surgery, yes)*-6’831.20 + (Revision or replacement of the hip joint without complicating diagnosis, yes)*-4’292.60 + (Tissue/cute transplantation, except in the hand, yes)*-4’703.50 + (Other spine surgeries, yes)*-8’423.30 + (Radiation therapy for diseases and disorders of the musculoskeletal system and connective tissue, yes)*-3’967.30 + (Other spine surgeries without complex surgery, yes)*-8’739.10 + (Other lower extremity and humerus surgeries or moderately complex elbow and forearm surgeries, yes)*-10’495.40 + (Diseases and disorders of the musculoskeletal system and connective tissue, yes)*-15’217.40 + (Osteomyelitis, yes)*-5’875.10 + (Malignant connective tissue neoplasm including pathological fracture, yes)*-6’559.70 + (Other connective tissue diseases, yes)*-7’422.40 + (Diseases and traumas of the spine, yes)*-6’531.20 + Bone Diseases and Arthropathies, yes)*-13’438.60 + (Muscle and tendon disease or sprain, yes)*-14’818.00 + (Tendinitis, myositis and bursitis, yes)*-6’256.00 + (Treatment for connective tissue diseases, yes)*-10’522.80 + (Forearm, wrist, hand, or foot trauma, yes)*-12’530.80 + (Severe trauma to the upper or lower extremity, yes)*-10’865.70 + (Other connective tissue diseases, yes)*-11’397.50 + (Moderate trauma of the upper or lower limb, yes)*-11’833.80 + (Diseases and disorders of the musculoskeletal system and connective tissue, yes)*42’539.90 + (Tumor endoprosthesis implantation or complex multi-stage procedure, yes)*17’283.90 + (Complex negative pressure therapy for diseases and disorders of the musculoskeletal system and connective tissue, yes)*28’082.80 + Tissue transplantation with microvascular anastomosis for skin diseases, yes)*11’108.80 + (Skin transplantation, flap surgery, extensive lymphadenectomy, tissue transplantation with microvascular anastomosis, yes)*9’843.20 + (Lower extremity skin surgery for ulcer or infection, yes)*1’181.20 + (Lower extremity skin procedures except for ulcer or infection, yes)*-9’861.30 + (Mastectomy with prosthetic implantation and plastic surgery for malignant neoplasms, yes)*423.6 + (Minor breast surgery with axillary lymphadenectomy, yes)*-8’485.60 + (Other skin transplantation or debridement with complex diagnostics, yes)*-527.3 + (Pilonidal and perianal interventions, yes)*-12’605.60 + (Plastic surgery on skin, subcutaneous tissue and breasts, yes)*-8’120.90 + (Other skin, subcutaneous tissue and breast surgeries, yes)*-10’578.10 + (Breast plastic surgery for malignant neoplasms with challenging reconstruction, yes)*982.5 + (Bilateral mastectomy for malignant neoplasms or radiation therapy with operative procedure for skin, subcutaneous tissue and breast diseases and disorders, yes)*2’041.40 + (Other radiation therapy for skin, subcutaneous tissue and breast diseases and disorders, yes)*-5’874.60 + (Other skin grafting or debridement with lymphadenectomy, yes)*-10’052.60 + (Other skin transplantation or debridement without complex surgery, yes)*-10’214.70 + (Major breast surgery for malignant neoplasms, yes)*-7’126.00 + (Breast surgeries except for malignant neoplasms with extensive surgery, yes)*-9’761.00 + (Minor breast surgeries for malignant neoplasms, yes)*-11’318.90 + (Skin ulcer, yes)*7’419.80 + (Severe skin diseases, yes)*-14’058.80 + (Breast plastic surgery with complex skin grafting, yes)*-6’803.50 + (Infection / inflammation of the skin and subcutaneous tissue, yes)*-11’134.90 + (Mild to moderate skin diseases, yes)*-11’281.40 + (Skin Diseases, yes)*-15’848.00 + (Adrenal surgeries for malignant neoplasms, yes)*3’274.20 + (Major obesity surgery with complex intervention, yes)*-1’538.70 + (Thyroid, parathyroid and thyroglossal duct surgeries, without parathyroidectomies, yes)*-9’128.30 + (Other procedures for endocrine, nutritional, and metabolic diseases, yes)*-301.1 + (Surgeries on the adrenals except for malignant neoplasms and extensive lymphadenectomy, yes)*-4’143.80 + (Radiation therapy for endocrine, nutritional and metabolic diseases, yes)*-5’642.40 + (Complex multi-stage procedures for endocrine, nutritional, and metabolic diseases, yes)*-5’314.30 + (Appendicitis unspecified, yes)*18’139.60 + (Diabetes mellitus and severe nutritional disorders, yes)*-10’780.70 + (Miscellaneous Metabolic Disorders on Paraplegia/Tetraplegia, yes)*-14’286.80 + (Congenital disorders of metabolism, yes)*-10’883.40 + (Surgical insertion of peritoneal dialysis catheter, yes)*-11’876.10 + (Kidney and ureter surgeries and major bladder surgeries for neoplasia, yes)*6’494.20 + (Kidney and ureter surgeries and major bladder surgeries except for neoplasia, yes)*-5’492.20 + (Transurethral prostatectomy, yes)*-11’381.10 + (Minor bladder surgery, yes)*-13’270.60 + (Complex urethral interventions, yes)*-12’852.30 + (Other surgeries for urinary tract diseases, yes)*-8’644.80 + (Bladder reconstruction and continent pocket for neoplasm without multivisceral surgery , yes)*23’137.20 + (Complex transurethral, percutaneous-transurethral interventions, yes)*-5’229.90 + (Radiotherapy for diseases and disorders of the urinary system, yes)*-14’126.50 + (Kidney and ureter surgeries and major bladder surgeries for neoplasia, yes)*-2’143.30 + (Other urethral surgeries, yes)*-14’088.20 + (Complex transurethral, percutaneous-transurethral, and other complex retroperitoneal surgeries, yes)*-13’131.30 + (Transurethral surgeries except prostatectomy and complex ureterorenoscopy with lithotripsy , yes)*-12’049.50 + (Transurethral surgeries except prostatectomy and complex ureterorenoscopies, yes)*-12’628.30 + (Complex multi-stage procedures for diseases and disorders of the urinary tract, yes)*17’289.70 + (Multivisceral intervention for diseases and disorders of the urinary organs, yes)*22’667.40 + (Complicating procedures with specific surgery or multivisceral intervention for diseases and disorders of the urinary tract, yes)*8’600.80 + (Diagnostic Ureterorenoscopy, yes)*-13’583.60 + (Extracorporeal shock wave lithotripsy for urinary calculosis, yes)*-13’473.40 + (Chronic renal disease, yes)*-3’903.80 + (Neoplasms of the urinary tract, yes)*-13’753.70 + (Urinary tract infections , yes)*-10’688.90 + (Urinary calculosis and urinary tract obstruction, yes)*-13’384.90 + (Other moderately severe diseases of the urinary system or bladder paralysis, yes)*-14’329.60 + (Other serious diseases of the urinary tract, yes)*-8’123.60 + (Diseases and disorders of the urinary system, yes)*-15’653.70 + (Renal failure with dialysis, yes)*-16’037.20 + (Major operations on the male pelvis, yes)*4’124.20 + (Transurethral prostatectomy, yes)*-10’971.30 + (Penis surgery, yes)*-12’702.40 + (Testicle surgery, yes)*-12’370.30 + (Circumcision and other penile procedures, yes)*-15’617.70 + (Other male genital procedures, yes)*-9’821.90 + (Male genital procedures for malignant neoplasms , yes)*-9’304.70 + (Radiation therapy for diseases and disorders of the male genital apparatus, yes)*2’422.60 + (Transurethral laser destruction of the prostate, yes)*-11’743.20 + (Major bowel or bladder surgeries for diseases and disorders of the male genital system, yes)*29’941.40 + (Malignant neoplasms of the male genital system, yes)*-14’042.70 + (Benign prostatic hyperplasia, yes)*-15’424.60 + (Infection / inflammation of the male genital system, yes)*-12’124.20 + (Other diseases of the male genital apparatus and male sterilization, yes)*-15’299.90 + (Pelvic evisceration in women and radical vulvectomy or specific lymphadenectomy, yes)*7’510.00 + (Interventions on uterus and adnexa for malignant neoplasms of the ovary or adnexa, yes)*-4’154.60 + (Interventions on uterus and adnexa for malignant neoplasms of other organs, yes)*-1’550.70 + (Hysterectomy except for malignant neoplasms, yes)*-5’536.40 + (Ovariectomy and complex tube surgeries except for malignant neoplasms, yes)*-8’413.10 + (Complex reconstructive surgery on the female genital apparatus, yes)*-9’025.10 + (Other uterine and adnexal surgeries except for malignant neoplasms, yes)*-8’955.50 + (Endoscopic procedures on the female genital apparatus, yes)*-8’452.10 + (Other vagina, cervix and vulva surgeries or brachytherapy for diseases and disorders of the female genital system, yes)*-12’888.50 + (Diagnostic scraping, hysteroscopy, sterilization, yes)*-14’184.90 + (Other procedures on the female genital apparatus with specific intervention , yes)*-1’721.70 + (Major operations on vagina, cervix and vulva, yes)*-11’508.80 + (Hysterectomy and pelvic floor plastics except for malignant neoplasms, yes)*-7’498.20 + (Radiation therapy for diseases and disorders of the female genital system, yes)*-12’875.40 + (Hysterectomy except for malignant neoplasms, yes)*-5’450.40 + (Other reconstructive procedures on the female genital apparatus, yes)*-12’037.90 + (Other uterine and adnexal surgeries except for malignant neoplasms, yes)*-10’402.20 + (Multi-stage surgeries or complicating procedures with specific surgery for diseases and disorders of the female genital tract, yes)*9’209.30 + (Major bowel or bladder surgeries for diseases and disorders of the female genital system, yes)*9’984.40 + (Malignant neoplasms of the female genital apparatus, yes)*-11’810.30 + (Infection and inflammation of the female genital tract, yes)*-14’369.80 + (Menstrual disorders and other diseases of the female genital system with complex diagnostics, yes)*-14’715.20 + (Caesarean section with multiple complicating diagnoses, yes)*-8’648.70 + (Vaginal delivery with complicated procedure, yes)*-7’949.40 + (Ectopic pregnancy, yes)*-8’551.00 + (Hospitalization after childbirth or abortion, yes)*-13’844.80 + (Cervical ostium cerclage and closure, yes)*-10’168.70 + (Abortion with dilatation and curettage, by aspiration or hysterotomy, yes)*-12’857.50 + (Vaginal delivery with multiple complicating diagnoses of which at least one is severe, yes)*-6’984.40 + (Hospitalization after childbirth or abortion without interventional procedure, yes)*-12’560.20 + (Abortion without dilation and curettage, by aspiration or hysterotomy, yes)*-8’533.30 + (Other preparative hospitalization with extremely serious or severe comorbidities or complex diagnostics, yes)*-13’479.80 + (Spleen surgery, yes)*-6’719.50 + (Miscellaneous procedures for blood diseases, yes)*-5’926.30 + (Minor interventions for blood diseases, yes)*-12’403.60 + (Diseases of the reticuloendothelial and immune systems and coagulation disorders, yes)*-14’909.90 + (Lymphoma and leukemia with major operative procedures, yes)*-4’845.00 + (Major or complex procedures for hematologic and solid neoplasms or lymphoma and leukemia , yes)*18’336.40 + (Other hematologic and solid neoplasms with other operative procedure, yes)*-7’386.30 + (Lymphoma and leukemia with specific procedure, yes)*-3’796.80 + (Other hematologic and solid neoplasms with major operative procedures, yes)*-4’035.50 + (Other hematologic and solid neoplasms with specific operative procedure, yes)*-8’881.40 + (Other hematologic and solid neoplasms without extremely serious or severe comorbidities, yes)*-13’317.80 + (Highly complex chemotherapy with surgery for hematologic and solid malignancies, yes)*36’973.70 + (Acute myeloid leukemia with intensive chemotherapy, yes)*-9’971.20 + (Lymphoma and non-acute leukemia with septicemia or with agranulocytosis , yes)*-3’966.30 + (Other hematologic and solid neoplasms with complicating diagnosis or dialysis , yes)*-8’388.80 + (Other acute leukemia with highly complex chemotherapy, yes)*-3’601.60 + (Hematological and solid neoplasms, yes)*-13’845.60 + (HIV disease with operative procedure, yes)*40’124.10 + (Infection on HIV disease with complex diagnostics, yes)*9’625.50 + (Other disorders on HIV disease with myocardial infarction or chronic ischemic heart disease, yes)*-8’839.10 + (Infectious and parasitic diseases with complex operative procedure, yes)*572.9 + (Complex intensive care or complex negative pressure therapy for infectious and parasitic diseases, yes)*30’072.40 + (Septicemia with complicating procedures or on organ transplant , yes)*9’042.90 + (Postoperative or post-traumatic infections with complicating procedures or complicating diagnosis, yes)*-10’870.90 + (Other diseases of viral origin, yes)*-10’567.80 + (Other infectious and parasite diseases, yes)*5’490.40 + (Other infectious and parasitic diseases, yes)*-3’346.70 + (Alcohol intoxication and withdrawal or abuse-induced disorders, yes)*-7’315.00 + (Polytrauma with assisted breathing or craniotomy, or complex negative pressure therapy , yes)*26’283.60 + (Polytrauma with specific interventions, complicating procedures, or interventions on multiple locations, yes)*11’443.90 + (Polytrauma without significant intervention , yes)*-4’539.70 + (Trauma reconstruction surgeries with complicating procedures, yes)*-4’285.80 + (Other operations for traumatisms of the lower limbs, yes)*-7’416.00 + (Other interventions for traumatisms of the hand, with complex intervention, yes)*-11’280.70 + (Other interventions for other traumatisms, yes)*-4’889.40 + (Reimplantation for traumatic amputation, with reimplantation of more than one toe or hand, yes)*-12’557.00 + (Traumatism and allergic reactions, yes)*-10’774.70 + (Poisonings and toxic effects of drugs, medications and other substances or consequences of medical treatment, yes)*-11’481.40 + (Other disease caused by trauma, poisoning or toxic effect, yes)*-14’690.30 + (Surgery or assisted breathing, yes)*-9’659.80 + (Other burns with skin graft on septicemia or with complicating procedures, yes)*-3’230.40 + (Other burns with other surgeries, yes)*-2’321.30 + (Other burns, yes)*-4’954.50 + (Operative procedures for other conditions requiring the use of health care services, yes)*-8’671.70 + (Other factors affecting health status and further treatment after completion of treatment, yes)*-15’497.30 + (Disorders, symptoms, other abnormalities, and further treatment with specific diagnosis and procedure or dialysis, yes)*6’090.90

**Supplemental appendix 2: Full regression model output of incremental cost (USD) impact of appropriate and inappropriate red cell transfusion on hospital costs after adjusting for confounding factors.**

| Variable | Estimate | Std. Error | P-value | Lower Confidence Level | Upper Confidence Level |
| --- | --- | --- | --- | --- | --- |
| (Intercept) | 13’275.2 | 759.1 | <0.001 | 11’787.3 | 14’763.1 |
| No RBC Transfusion | Reference | | | | |
| Appropriate RBC transfusion | 12’260.2 | 243.6 | <0.001 | 11’782.7 | 12’737.7 |
| Inappropriate RBC transfusion | 9’778.6 | 214.3 | <0.001 | 9’358.3 | 10’198.6 |
| AGE, years | 47.0 | 2.0 | <0.001 | 43.0 | 51.0 |
| Sex, female | 285.9 | 66.3 | <0.001 | 155.9 | 415.9 |
| ASA Class | 1’273.2 | 47.4 | <0.001 | 1’180.4 | 1’366.0 |
| Oncology diagnosis (yes/no) | 1’982.8 | 166.3 | <0.001 | 1’656.8 | 2’308.7 |
| Platelet transfusion (yes/no) | 1’610.1 | 577.9 | 0.005 | 477.4 | 2’742.8 |
| Plasma transfusion (yes/no) | 4’113.5 | 668.0 | <0.001 | 2’804.1 | 5’422.8 |
| Wound debridement for injuries with major complication or comorbidity | Reference | | | | |
| Wound debridement for injuries with complication or comorbidity | -5’640.50 | 1’705.40 | <0.001 | -8’983.10 | -2’297.90 |
| Prolonged assisted ventilation | 36’079.90 | 4’788.60 | <0.001 | 26’694.10 | 45’465.70 |
| Assisted ventilation | 36’593.40 | 1’581.80 | <0.001 | 33’493.10 | 39’693.80 |
| Bone marrow transplant | 16’544.50 | 6’730.80 | 0.014 | 3’352.00 | 29’737.00 |
| Complex intensive care unit | 33’416.60 | 1’489.60 | <0.001 | 30’496.90 | 36’336.30 |
| Complex negative pressure treatment | 33’801.40 | 3’426.90 | <0.001 | 27’084.50 | 40’518.20 |
| Transplant rejection | -9’653.40 | 2’149.80 | <0.001 | -13’867.10 | -5’439.70 |
| Radiotherapy | 30’106.70 | 2’831.00 | <0.001 | 24’557.80 | 35’655.60 |
| Acute geriatric rehabilitation | 11’348.40 | 955.4 | <0.001 | 9’475.80 | 13’221.00 |
| Complex palliative medicine treatment | 20’563.30 | 1’508.80 | <0.001 | 17’606.00 | 23’520.60 |
| Complex OR procedures for diseases and disorders of the nervous system | 31’593.30 | 1’448.30 | <0.001 | 28’754.60 | 34’432.00 |
| Complex craniotomy or spine surgery | 19’710.70 | 1’058.40 | <0.001 | 17’636.20 | 21’785.10 |
| Operations for para- or quadriplegia, brain disease, and interventions for cerebral palsy, muscular dystrophy, neuropathy, or operations on the spine and spinal cord surgery | 395.6 | 1’353.30 | 0.77 | -2’257.00 | 3’048.10 |
| Extracranial vessel surgery, ASD closure or cardiac pacemaker closure | -2’016.10 | 869.4 | 0.02 | -3’720.10 | -312.1 |
| Decompression for carpal or tarsal tunnel syndrome | -15’841.60 | 919.3 | <0.001 | -17’643.50 | -14’039.80 |
| Rubella with neurological complications | -6’037.90 | 3’082.70 | 0.05 | -12’079.90 | 4.2 |
| Other cranial surgery | 4’159.70 | 1’471.40 | 0.005 | 1’275.70 | 7’043.60 |
| Radiotherapy for diseases and disorders of the nervous system | 12’988.80 | 4’788.30 | 0.007 | 3’603.60 | 22’373.90 |
| Peripheral and cranial nerve surgery or complex skin surgery or implant removal | -12’261.80 | 837.9 | <0.001 | -13’904.00 | -10’619.50 |
| Chronic viral hepatitis | 1’607.90 | 1’226.80 | 0.19 | -796.7 | 4’012.50 |
| Craniotomy or major spine surgery | 8’849.00 | 799 | <0.001 | 7’283.00 | 10’415.10 |
| Neurostimulator implant | -9’313.70 | 1’608.40 | <0.001 | -12’466.20 | -6’161.20 |
| Drug infusion pump implant | -11’848.80 | 3’933.50 | 0.003 | -19’558.40 | -4’139.10 |
| Nervous system diseases and disorders with complex critical care | 40’346.50 | 2’242.60 | <0.001 | 35’950.90 | 44’742.10 |
| Complex neurologic treatment of acute stroke with surgery | 12’638.10 | 1’151.30 | <0.001 | 10’381.60 | 14’894.60 |
| Specific acute diseases and traumas of the spinal cord | 14’534.20 | 1’530.80 | <0.001 | 11’533.90 | 17’534.50 |
| Dementia and other chronic disorders of brain function | -4’047.40 | 6’730.90 | 0.548 | -17’239.90 | 9’145.20 |
| Delirium | -5’387.70 | 6’731.00 | 0.423 | -18’580.50 | 7’805.10 |
| Neoplasms of the nervous system or stupor and coma of non-traumatic origin | -16’526.10 | 3’427.50 | <0.001 | -23’243.90 | -9’808.20 |
| Transient cerebral ischemia and occlusion of extracranial vessels | -8’820.50 | 6’730.90 | 0.19 | -22’013.10 | 4’372.10 |
| Apoplexy | 10’743.30 | 3’933.80 | 0.006 | 3’033.00 | 18’453.60 |
| Cranial and peripheral nerve diseases | -12’008.40 | 4’788.50 | 0.012 | -21’393.90 | -2’622.90 |
| Nervous system infection except viral meningitis | -1’016.90 | 4’788.50 | 0.832 | -10’402.30 | 8’368.50 |
| Epileptic seizures or video-EEG or complex epilepsy treatment | -8’024.80 | 6’730.90 | 0.233 | -21’217.40 | 5’167.70 |
| Cephalea | -12’853.90 | 6’730.90 | 0.056 | -26’046.50 | 338.7 |
| Intracranial trauma | 2’717.40 | 4’788.50 | 0.57 | -6’668.00 | 12’102.90 |
| Vascular myelopathies or other diseases of the nervous system | 9’775.40 | 6’730.80 | 0.146 | -3’417.00 | 22’967.90 |
| Degenerative diseases of the nervous system or cerebral palsy | -2’781.70 | 2’830.40 | 0.326 | -8’329.40 | 2’766.00 |
| Orbit and ocular bulb surgeries | -11’937.50 | 6’731.10 | 0.076 | -25’130.50 | 1’255.50 |
| Eyelid surgery | -11’066.50 | 4’788.50 | 0.021 | -20’452.00 | -1’680.90 |
| Interventions on the lacrimal apparatus | -9’203.90 | 1’045.20 | <0.001 | -11’252.50 | -7’155.30 |
| Conjunctive surgeries or other procedures | -8’354.50 | 3’426.60 | 0.015 | -15’070.60 | -1’638.40 |
| Malignant Tumor: Stomach Body | -3’367.70 | 3’933.20 | 0.392 | -11’076.70 | 4’341.40 |
| Malignant cancer of the rectum | -11’656.80 | 3’082.70 | <0.001 | -17’698.90 | -5’614.60 |
| Bimaxillary osteotomy and complex jaw surgery | 5’443.30 | 3’083.30 | 0.078 | -600 | 11’486.60 |
| Complex salivary gland operations | -8’633.90 | 925.9 | <0.001 | -10’448.60 | -6’819.30 |
| Challenging surgeries on sinuses, mastoid, middle ear and other procedures on salivary glands | -9’913.30 | 772.6 | <0.001 | -11’427.70 | -8’399.00 |
| Oral cavity and mouth surgery for malignant neoplasm | -12’671.30 | 1’508.00 | <0.001 | -15’626.90 | -9’715.70 |
| Tonsillectomy for malignant neoplasm or miscellaneous ear, nose, mouth and throat | -14’187.30 | 2’242.60 | <0.001 | -18’582.70 | -9’791.90 |
| Other ear, nose, mouth, and throat surgeries | -11’830.00 | 963.8 | <0.001 | -13’719.10 | -9’940.90 |
| Tonsillectomy or adenectomy except for malignant neoplasm | -12’102.70 | 3’426.60 | <0.001 | -18’818.80 | -5’386.60 |
| Tracheostomy or irradiation session for multidrug-resistant pathogen | 15’937.30 | 1’508.50 | <0.001 | 12’980.70 | 18’893.90 |
| Removal of osteosynthesis material from jaw and face | -15’363.50 | 4’788.50 | 0.001 | -24’748.90 | -5’978.10 |
| Oral cavity and mouth surgeries except for malignant neoplasm | -14’334.40 | 1’381.50 | <0.001 | -17’042.20 | -11’626.50 |
| Complex skin plastics and major head and neck surgeries | 8’282.20 | 1’486.60 | <0.001 | 5’368.40 | 11’196.00 |
| Moderately complex head and neck surgeries, radiation therapy with surgery | -11’519.90 | 1’429.80 | <0.001 | -14’322.30 | -8’717.60 |
| Single-maxillary osteotomy and complex head and neck or other surgery on the head and neck for malignant neoplasm | -4’819.80 | 1’608.60 | 0.003 | -7’972.60 | -1’666.90 |
| Jaw surgery and other head and neck surgery except for malignant neoplasia | -13’106.00 | 1’705.30 | <0.001 | -16’448.50 | -9’763.50 |
| Ear, nose, mouth, and throat surgeries except for malignant neoplasm malignant, without plastic surgery of the buccal floor | -13’761.60 | 777.1 | <0.001 | -15’284.70 | -12’238.40 |
| Nose and sinus surgeries for malignant neoplasia | -3’474.00 | 2’830.60 | 0.22 | -9’021.90 | 2’073.90 |
| Diseases and disorders of the ear, nose, mouth, and throat with complex intensive therapy | 13’745.60 | 4’788.40 | 0.004 | 4’360.20 | 23’130.90 |
| Complex and very complex operations on the nose | -11’860.70 | 770.6 | <0.001 | -13’371.10 | -10’350.30 |
| Other nose surgeries | -13’607.90 | 1’448.80 | <0.001 | -16’447.50 | -10’768.30 |
| Tooth extraction and repair | -16’935.60 | 1’367.40 | <0.001 | -19’615.80 | -14’255.50 |
| Malignant neoplasms of the ear, nose, mouth and throat | -15’211.00 | 1’042.20 | <0.001 | -17’253.70 | -13’168.40 |
| Epistaxis or otitis media or upper airway infections | -9’917.80 | 2’069.30 | <0.001 | -13’973.70 | -5’861.90 |
| Laryngotracheitis and epiglottitis | -12’323.90 | 6’730.80 | 0.067 | -25’516.50 | 868.6 |
| Traumatism and deformities of the nose | -11’826.70 | 6’730.90 | 0.079 | -25’019.20 | 1’365.90 |
| Other diseases of the ear, nose, mouth, and throat | -15’051.80 | 1’243.80 | <0.001 | -17’489.70 | -12’614.00 |
| Diseases of the teeth and oral cavity without extraction and repair of teeth | -13’541.60 | 2’635.60 | <0.001 | -18’707.30 | -8’375.70 |
| Revision surgery, bilateral lobectomy | 8’807.80 | 1’999.50 | <0.001 | 4’888.70 | 12’726.80 |
| Other respiratory procedures or major bronchus surgery | -4’076.30 | 936.5 | <0.001 | -5’911.80 | -2’240.80 |
| Other major chest surgery or extracorporeal circulation | 4’676.90 | 820.1 | <0.001 | 3’069.50 | 6’284.30 |
| Other lung resection, thoracic organ biopsy, and chest wall surgery thoracic wall | -5’484.60 | 834.6 | <0.001 | -7’120.40 | -3’848.80 |
| Interventions for sleep apnea | -14’454.40 | 6’730.90 | 0.032 | -27’647.00 | -1’261.80 |
| Radiotherapy for respiratory diseases and disorders | -13’868.00 | 6’730.80 | 0.039 | -27’060.50 | -675.5 |
| Respiratory diseases and disorders with complex intensive care unit | 24’102.50 | 1’508.40 | <0.001 | 21’146.10 | 27’058.90 |
| Malnutrition | 17’354.40 | 3’426.90 | <0.001 | 10’637.60 | 24’071.20 |
| Respiratory insufficiency or pulmonary embolism | -13’840.50 | 6’731.20 | 0.04 | -27’033.70 | -647.3 |
| Chronic obstructive pulmonary disease | -4’638.40 | 3’933.20 | 0.238 | -12’347.50 | 3’070.70 |
| Severe chest trauma | -4’992.10 | 6’730.90 | 0.458 | -18’184.60 | 8’200.50 |
| Acute bronchitis | -16’393.00 | 6’730.90 | 0.015 | -29’585.50 | -3’200.50 |
| Respiratory tract neoplasms | -5’239.80 | 1’429.90 | <0.001 | -8’042.50 | -2’437.20 |
| Pleural effusion | 9’082.30 | 6’730.80 | 0.177 | -4’110.20 | 22’274.80 |
| Interstitial lung disease | -5’260.60 | 4’788.30 | 0.272 | -14’645.70 | 4’124.60 |
| Other respiratory diseases | -10’332.00 | 2’830.50 | <0.001 | -15’879.80 | -4’784.20 |
| Tuberculosis or pneumothorax | -4’885.00 | 2’351.00 | 0.038 | -9’493.00 | -277.1 |
| Other respiratory tract infections and inflammations | 8’696.40 | 1’832.20 | <0.001 | 5’105.30 | 12’287.50 |
| Control and optimization of a pre-existing artificial respiration therapy | -15’325.60 | 6’730.80 | 0.023 | -28’518.00 | -2’133.20 |
| Challenging multi-time procedures or complex negative pressure therapy | 18’372.70 | 2’244.80 | <0.001 | 13’972.80 | 22’772.60 |
| Reconstructive vascular procedures or thoracoabdominal aneurysm with complex aortic procedure | 5’609.60 | 2’674.20 | 0.036 | 368.2 | 10’851.00 |
| Cardiac pacemaker implantation | -3’427.00 | 1’017.20 | <0.001 | -5’420.80 | -1’433.20 |
| Amputation for circulatory disease of the upper extremity and toes for diabetes mellitus with complications | 10.1 | 930.9 | 0.991 | -1’814.40 | 1’834.70 |
| Complex or multiple vascular interventions | -11.6 | 1’105.60 | 0.992 | -2’178.50 | 2’155.40 |
| Cardiac pacemaker replacement | -6’569.30 | 2’350.90 | 0.005 | -11’177.00 | -1’961.60 |
| Revision of cardiac pacemaker or automatic defibrillator | -12’851.70 | 3’426.70 | <0.001 | -19’568.00 | -6’135.40 |
| Bilateral ligation and stripping of veins with ulceration | -9’229.70 | 6’730.80 | 0.17 | -22’422.20 | 3’962.80 |
| Other procedures for diseases of the cardiovascular system | -3’901.10 | 987.5 | <0.001 | -5’836.70 | -1’965.60 |
| Amputation for circulatory diseases except upper limb and toes | 14’641.20 | 1’141.60 | <0.001 | 12’403.60 | 16’878.70 |
| Reconstructive vascular procedures | 4’815.30 | 933.9 | <0.001 | 2’984.90 | 6’645.80 |
| Other major reconstructive vascular procedures | 591.3 | 846.8 | 0.485 | -1’068.40 | 2’251.00 |
| Other cardiothoracic procedures | -1’021.80 | 4’788.50 | 0.831 | -10’407.30 | 8’363.70 |
| Diseases and disorders of the cardiovascular system with complex critical care | 25’370.70 | 2’481.90 | <0.001 | 20’506.20 | 30’235.20 |
| Other Behavior Disorders | -716 | 2’149.80 | 0.739 | -4’929.80 | 3’497.70 |
| Vein ligation and stripping | -12’686.80 | 800.3 | <0.001 | -14’255.40 | -11’118.30 |
| Invasive cardiologic diagnostics for acute myocardial infarction | -1’591.90 | 6’735.20 | 0.813 | -14’793.00 | 11’609.10 |
| Vegetative dysfunction of the cardiovascular system | 31’273.50 | 6’730.90 | <0.001 | 18’080.90 | 44’466.00 |
| Endovascular stent insertion in the aorta | 3’050.30 | 916.1 | <0.001 | 1’254.80 | 4’845.80 |
| Psychological and behavioral factors associated with disorders or diseases | -4’425.00 | 1’045.10 | <0.001 | -6’473.40 | -2’376.50 |
| Complex or moderately complex vascular interventions | -4’658.80 | 889 | <0.001 | -6’401.30 | -2’916.40 |
| Acute myocardial infarction without invasive cardiological diagnostics | 8’358.30 | 4’788.30 | 0.081 | -1’026.70 | 17’743.40 |
| Endocarditis | -760.9 | 3’933.30 | 0.847 | -8’470.30 | 6’948.50 |
| Heart failure and shock | 4’774.50 | 2’242.60 | 0.033 | 379 | 9’170.00 |
| Venous Thrombosis | -6’055.30 | 6’731.20 | 0.368 | -19’248.50 | 7’138.00 |
| Skin Ulcer on Circulatory Diseases | -2’786.00 | 3’934.30 | 0.479 | -10’497.20 | 4’925.20 |
| Peripheral vascular diseases | 13’871.80 | 6’735.00 | 0.039 | 671.1 | 27’072.50 |
| Arterial Hypertension | -4’682.20 | 6’730.90 | 0.487 | -17’874.90 | 8’510.50 |
| Moderate mental impairment | -9’200.40 | 4’788.30 | 0.055 | -18’585.50 | 184.7 |
| Severe mental impairment | -12’540.20 | 6’730.80 | 0.062 | -25’732.80 | 652.2 |
| Very severe mental impairment | -10’908.90 | 3’933.30 | 0.006 | -18’618.30 | -3’199.60 |
| Other diseases of the cardiovascular system | -11’683.60 | 1’227.10 | <0.001 | -14’088.80 | -9’278.40 |
| Complicated surgery or diagnosis on intestine | 7’901.50 | 1’017.50 | <0.001 | 5’907.10 | 9’895.90 |
| Major operations on stomach, esophagus and duodenum with highly complex surgery | 16’034.60 | 1’510.70 | <0.001 | 13’073.60 | 18’995.60 |
| Minor interventions on small intestine and colon | 5’731.30 | 1’146.00 | <0.001 | 3’485.00 | 7’977.50 |
| Appendectomy for peritonitis | -10’027.80 | 884.4 | <0.001 | -11’761.20 | -8’294.40 |
| Complex reconstruction of the abdominal wall | -7’708.90 | 823.2 | <0.001 | -9’322.40 | -6’095.40 |
| Hernia surgery | -12’080.00 | 764.1 | <0.001 | -13’577.70 | -10’582.30 |
| Specific interventions on the hepatobiliary system, pancreas, spleen | 2’336.70 | 1’881.80 | 0.214 | -1’351.70 | 6’025.10 |
| Pyloromyotomy or anoproctoplasty and reconstruction of anus and sphincter | -10’225.10 | 1’555.20 | <0.001 | -13’273.30 | -7’176.90 |
| Other digestive tract procedures with complex procedure | -5’152.30 | 939.5 | <0.001 | -6’993.80 | -3’310.90 |
| Other bowel surgery or enterostomy | 19’110.40 | 1’080.70 | <0.001 | 16’992.20 | 21’228.70 |
| Complex rectal resection or pelvic evisceration or surgery for malignant neoplasm | 12’039.00 | 1’145.60 | <0.001 | 9’793.60 | 14’284.30 |
| Other rectal resection without specific intervention | 3’718.90 | 904.3 | <0.001 | 1’946.50 | 5’491.30 |
| Small bowel and colon or other surgery on the stomach, esophagus, and duodenum | -1’633.00 | 773 | 0.035 | -3’148.00 | -117.9 |
| Other stomach, esophagus, and duodenum surgeries for malignant neoplasm | -2’767.50 | 811.1 | <0.001 | -4’357.30 | -1’177.70 |
| Surgery for adhesions | -8’600.20 | 967 | <0.001 | -10’495.60 | -6’704.90 |
| Appendectomy on peritonitis | -10’317.60 | 912.4 | <0.001 | -12’106.00 | -8’529.30 |
| Appendectomy except on peritonitis | -11’231.00 | 876.5 | <0.001 | -12’949.00 | -9’512.90 |
| Abdominal wall, umbilical and other hernia surgeries | -12’911.40 | 776.6 | <0.001 | -14’433.60 | -11’389.20 |
| Inguinal and femoral hernia surgery | -12’510.50 | 868.1 | <0.001 | -14’212.10 | -10’808.90 |
| Other anus surgeries | -13’243.50 | 783.9 | <0.001 | -14’780.00 | -11’707.10 |
| Other radiation therapy for diseases and disorders of the digestive system | 36’298.60 | 6’733.20 | <0.001 | 23’101.40 | 49’495.80 |
| Complex multi-stage procedures for digestive diseases and disorders | 4’766.10 | 3’428.20 | 0.164 | -1’953.10 | 11’485.30 |
| Complex negative pressure therapy for digestive diseases and disorders | 32’668.80 | 4’788.70 | <0.001 | 23’282.90 | 42’054.70 |
| Multivisceral surgery for diseases and disorders of the digestive system | 22’620.80 | 2’242.50 | <0.001 | 18’225.40 | 27’016.20 |
| Complicating procedures or complex intensive care with specific surgery for diseases and disorders of the digestive system | 25’526.80 | 1’584.60 | <0.001 | 22’421.00 | 28’632.60 |
| Gastroscopy of various types for severe digestive diseases associated with complicating procedures | -7’546.70 | 2’351.30 | 0.001 | -12’155.30 | -2’938.10 |
| Other gastroscopy for serious diseases of the digestive system | -10’720.00 | 4’789.00 | 0.025 | -20’106.50 | -1’333.40 |
| Colonoscopy or complicating surgery | 1’682.90 | 2’479.30 | 0.497 | -3’176.60 | 6’542.40 |
| Malignant neoplasm of the digestive system | -9’882.00 | 1’253.90 | <0.001 | -12’339.60 | -7’424.40 |
| Inflammatory bowel disease or other serious disease of the digestive system | 2’639.50 | 3’082.80 | 0.392 | -3’402.90 | 8’681.90 |
| Digestive tract obstruction | -13’196.90 | 6’731.20 | 0.05 | -26’390.10 | -3.6 |
| Digestive diseases or gastrointestinal bleeding | -1’386.80 | 2’242.30 | 0.536 | -5’781.80 | 3’008.20 |
| Other severe diseases of the digestive system | -11’579.50 | 3’426.40 | <0.001 | -18’295.30 | -4’863.70 |
| Other digestive diseases of moderate severity | -14’153.20 | 1’129.50 | <0.001 | -16’367.00 | -11’939.30 |
| Other mild to moderate digestive diseases or abdominal pain | -12’750.00 | 2’635.50 | <0.001 | -17’915.50 | -7’584.50 |
| Gastrointestinal bleeding or peptic ulcer | -5’167.20 | 6’730.90 | 0.443 | -18’359.80 | 8’025.40 |
| Pancreas and liver surgery with complex surgery | 18’452.00 | 979.3 | <0.001 | 16’532.50 | 20’371.50 |
| Complex gallbladder and biliary tract surgeries | 13’251.50 | 1’640.00 | <0.001 | 10’037.20 | 16’465.90 |
| Laparotomy and moderately complex gallbladder and biliary tract surgery | 1’436.60 | 1’235.40 | 0.245 | -984.7 | 3’858.00 |
| Other hepatobiliary and pancreas procedures | -9’733.40 | 925.1 | <0.001 | -11’546.70 | -7’920.20 |
| Cholecystectomy with complex diagnostic | -10’084.30 | 784.4 | <0.001 | -11’621.70 | -8’546.80 |
| Laparoscopic cholecystectomy with complex diagnostic | -10’859.90 | 769.3 | <0.001 | -12’367.70 | -9’352.20 |
| Pancreas and liver surgeries and portosystemic shunts | 6’302.10 | 1’019.60 | <0.001 | 4’303.60 | 8’300.70 |
| Miscellaneous hepatobiliary surgeries with extremely serious complications | 1’586.70 | 1’430.20 | 0.267 | -1’216.40 | 4’389.80 |
| Complex multi-stage procedures for hepatobiliary and pancreatic diseases and disorders | 12’334.10 | 3’933.70 | 0.002 | 4’624.10 | 20’044.10 |
| Complex therapeutic ERCP | -3’238.50 | 1’024.80 | 0.002 | -5’247.20 | -1’229.80 |
| Liver cirrhosis and non-infectious hepatitis | -6’218.90 | 4’798.40 | 0.195 | -15’623.80 | 3’186.00 |
| Malignant neoplasm of the hepatobiliary system and pancreas | -8’035.20 | 1’357.90 | <0.001 | -10’696.70 | -5’373.70 |
| Diseases of the pancreas except malignant neoplasms with acute pancreatitis or liver cirrhosis | -14’428.40 | 6’730.90 | 0.032 | -27’621.00 | -1’235.70 |
| Liver diseases except malignant neoplasms, liver cirrhosis, and noninfectious hepatitis | -7’775.80 | 4’788.50 | 0.104 | -17’161.40 | 1’609.80 |
| Gallbladder and biliary tract diseases with extremely serious complications | -12’613.20 | 3’933.10 | 0.001 | -20’322.20 | -4’904.30 |
| Challenging Tissue/Skin Transplantation | 22’579.10 | 1’449.40 | <0.001 | 19’738.20 | 25’419.90 |
| Hip revision or replacement with complicating diagnosis | 2’299.10 | 942.3 | 0.015 | 452.1 | 4’146.00 |
| Knee revision or replacement with complicating diagnosis | 5’120.10 | 1’785.50 | 0.004 | 1’620.40 | 8’619.80 |
| Other major joint replacement or revision or hip replacement | -4’502.70 | 820.3 | <0.001 | -6’110.50 | -2’894.90 |
| Complex vertebral fusion | 15’249.90 | 1’165.50 | <0.001 | 12’965.40 | 17’534.30 |
| Amputation | 8’067.60 | 1’341.70 | <0.001 | 5’438.00 | 10’697.30 |
| Other multiple hip and femur surgeries | -3’744.30 | 793.7 | <0.001 | -5’300.00 | -2’188.60 |
| Vertebral fusion | -378.1 | 778.7 | 0.627 | -1’904.40 | 1’148.20 |
| Other spine surgeries | -9’039.60 | 762.6 | <0.001 | -10’534.20 | -7’544.90 |
| Limb lengthening surgery | -3’439.20 | 4’788.50 | 0.473 | -12’824.60 | 5’946.20 |
| Infection / inflammation of bones and joints | 2’525.90 | 974.2 | 0.01 | 616.4 | 4’435.40 |
| Multiple surgeries on humerus, tibia, fibula, and ankle | -5’665.30 | 764.2 | <0.001 | -7’163.10 | -4’167.40 |
| Amputation stump revision | -3’412.40 | 1’304.00 | 0.009 | -5’968.30 | -856.5 |
| Cranial and facial bone surgeries | -1’791.10 | 1’367.30 | 0.19 | -4’471.00 | 888.9 |
| Other shoulder or clavicle surgeries | -10’691.70 | 850.7 | <0.001 | -12’359.10 | -9’024.30 |
| Facial bone surgeries | -9’638.40 | 1’608.50 | <0.001 | -12’791.10 | -6’485.70 |
| Arthroscopy including biopsy or other bone or joint surgery | -12’460.20 | 770.1 | <0.001 | -13’969.60 | -10’950.80 |
| Vertebral body fusions | 5’988.50 | 1’039.30 | <0.001 | 3’951.40 | 8’025.60 |
| Multiple highly complex foot surgeries | -9’879.00 | 779 | <0.001 | -11’405.80 | -8’352.10 |
| Specific hip and femur or complex elbow and forearm surgeries | -11’111.90 | 777.4 | <0.001 | -12’635.70 | -9’588.10 |
| Tissue/skin transplant with complicating procedures or surgery on multiple locations | 5’623.80 | 2’069.30 | 0.007 | 1’567.90 | 9’679.60 |
| Local excision and removal of osteosynthesis material | -13’090.70 | 790.4 | <0.001 | -14’640.00 | -11’541.50 |
| Diagnostic procedures on bones and joints, including biopsy | -6’245.90 | 2’242.40 | 0.005 | -10’641.00 | -1’850.80 |
| Soft tissue surgeries for malignant neoplasms | -10’539.70 | 784.4 | <0.001 | -12’077.10 | -9’002.30 |
| Other bone or connective tissue surgeries | -967.7 | 995.6 | 0.331 | -2’919.10 | 983.8 |
| Complex shoulder surgeries | -10’785.00 | 754.1 | <0.001 | -12’263.10 | -9’307.00 |
| Complex knee surgeries | -8’086.30 | 806.8 | <0.001 | -9’667.60 | -6’505.00 |
| Complex multiple elbow and forearm surgeries | -7’723.80 | 921.9 | <0.001 | -9’530.70 | -5’916.90 |
| Wrist and hand surgeries with complex or moderately complex multi-stage surgery | -10’351.80 | 767.9 | <0.001 | -11’856.90 | -8’846.70 |
| Rehabilitation treatment for diseases and disorders of the musculoskeletal system and connective tissue | 21’840.90 | 4’788.60 | <0.001 | 12’455.20 | 31’226.70 |
| Bilateral hip or knee implantation | 1’758.60 | 2’830.60 | 0.534 | -3’789.40 | 7’306.60 |
| Infusions for Musculoskeletal Disorders | -2’893.80 | 3’083.30 | 0.348 | -8’937.10 | 3’149.50 |
| Knee endoprosthesis implantation with prosthesis replacement | -4’006.80 | 770.2 | <0.001 | -5’516.40 | -2’497.10 |
| Implant in knee endoprosthesis component replacement | -6’970.70 | 914.4 | <0.001 | -8’762.90 | -5’178.60 |
| Implantation and replacement of disc prosthesis, more than one segment | -9’810.00 | 6’730.90 | 0.145 | -23’002.50 | 3’382.60 |
| Implantation, replacement or revision of a hip prosthesis with complex surgery | -6’831.20 | 772.1 | <0.001 | -8’344.50 | -5’317.80 |
| Revision or replacement of the hip joint without complicating diagnosis | -4’292.60 | 779.4 | <0.001 | -5’820.30 | -2’764.90 |
| Tissue/cute transplantation, except in the hand | -4’703.50 | 1’785.10 | 0.008 | -8’202.40 | -1’204.70 |
| Other spine surgeries | -8’423.30 | 804.3 | <0.001 | -9’999.80 | -6’846.80 |
| Radiation therapy for diseases and disorders of the musculoskeletal system and connective tissue | -3’967.30 | 3’426.40 | 0.247 | -10’683.20 | 2’748.50 |
| Other spine surgeries without complex surgery | -8’739.10 | 6’731.20 | 0.194 | -21’932.20 | 4’454.10 |
| Other lower extremity and humerus surgeries or moderately complex elbow and forearm surgeries | -10’495.40 | 1’076.80 | <0.001 | -12’606.00 | -8’384.70 |
| Diseases and disorders of the musculoskeletal system and connective tissue | -15’217.40 | 4’788.30 | 0.001 | -24’602.50 | -5’832.20 |
| Osteomyelitis | -5’875.10 | 2’479.40 | 0.018 | -10’734.80 | -1’015.40 |
| Malignant connective tissue neoplasm including pathological fracture | -6’559.70 | 2’479.50 | 0.008 | -11’419.50 | -1’699.80 |
| Other connective tissue diseases | -7’422.40 | 2’479.30 | 0.003 | -12’281.90 | -2’563.00 |
| Diseases and traumas of the spine | -6’531.20 | 2’149.80 | 0.002 | -10’744.80 | -2’317.50 |
| Bone Diseases and Arthropathies | -13’438.60 | 2’830.40 | <0.001 | -18’986.20 | -7’890.80 |
| Muscle and tendon disease or sprain | -14’818.00 | 3’426.80 | <0.001 | -21’534.60 | -8’101.30 |
| Tendinitis, myositis and bursitis | -6’256.00 | 3’933.20 | 0.112 | -13’965.20 | 1’453.10 |
| Treatment for connective tissue diseases | -10’522.80 | 1’830.30 | <0.001 | -14’110.20 | -6’935.50 |
| Forearm, wrist, hand, or foot trauma | -12’530.80 | 2’830.70 | <0.001 | -18’078.90 | -6’982.60 |
| Severe trauma to the upper or lower extremity | -10’865.70 | 2’149.80 | <0.001 | -15’079.30 | -6’652.00 |
| Other connective tissue diseases | -11’397.50 | 1’638.30 | <0.001 | -14’608.70 | -8’186.40 |
| Moderate trauma of the upper or lower limb | -11’833.80 | 3’933.70 | 0.003 | -19’543.90 | -4’123.80 |
| Diseases and disorders of the musculoskeletal system and connective tissue | 42’539.90 | 6’731.30 | <0.001 | 29’346.50 | 55’733.40 |
| Tumor endoprosthesis implantation or complex multi-stage procedure | 17’283.90 | 3’086.00 | <0.001 | 11’235.30 | 23’332.50 |
| Complex negative pressure therapy for diseases and disorders of the musculoskeletal system and connective tissue | 28’082.80 | 1’936.90 | <0.001 | 24’286.50 | 31’879.10 |
| Tissue transplantation with microvascular anastomosis for skin diseases | 11’108.80 | 1’072.90 | <0.001 | 9’006.00 | 13’211.60 |
| Skin transplantation, flap surgery, extensive lymphadenectomy, tissue transplantation with microvascular anastomosis | 9’843.20 | 1’080.10 | <0.001 | 7’726.20 | 11’960.30 |
| Lower extremity skin surgery for ulcer or infection | 1’181.20 | 1’119.10 | 0.291 | -1’012.40 | 3’374.70 |
| Lower extremity skin procedures except for ulcer or infection | -9’861.30 | 1’397.00 | <0.001 | -12’599.40 | -7’123.20 |
| Mastectomy with prosthetic implantation and plastic surgery for malignant neoplasms | 423.6 | 937.1 | 0.651 | -1’413.20 | 2’260.40 |
| Minor breast surgery with axillary lymphadenectomy | -8’485.60 | 880.1 | <0.001 | -10’210.70 | -6’760.60 |
| Other skin transplantation or debridement with complex diagnostics | -527.3 | 1’413.20 | 0.709 | -3’297.00 | 2’242.50 |
| Pilonidal and perianal interventions | -12’605.60 | 946.3 | <0.001 | -14’460.40 | -10’750.80 |
| Plastic surgery on skin, subcutaneous tissue and breasts | -8’120.90 | 873.1 | <0.001 | -9’832.10 | -6’409.70 |
| Other skin, subcutaneous tissue and breast surgeries | -10’578.10 | 821.5 | <0.001 | -12’188.10 | -8’968.00 |
| Breast plastic surgery for malignant neoplasms with challenging reconstruction | 982.5 | 1’880.70 | 0.601 | -2’703.80 | 4’668.70 |
| Bilateral mastectomy for malignant neoplasms or radiation therapy with operative procedure for skin, subcutaneous tissue and breast diseases and disorders | 2’041.40 | 1’055.20 | 0.053 | -26.9 | 4’109.70 |
| Other radiation therapy for skin, subcutaneous tissue and breast diseases and disorders | -5’874.60 | 1’397.10 | <0.001 | -8’612.90 | -3’136.20 |
| Other skin grafting or debridement with lymphadenectomy | -10’052.60 | 981.7 | <0.001 | -11’976.70 | -8’128.50 |
| Other skin transplantation or debridement without complex surgery | -10’214.70 | 991.7 | <0.001 | -12’158.50 | -8’270.90 |
| Major breast surgery for malignant neoplasms | -7’126.00 | 775.5 | <0.001 | -8’646.00 | -5’606.00 |
| Breast surgeries except for malignant neoplasms with extensive surgery | -9’761.00 | 840.7 | <0.001 | -11’408.80 | -8’113.20 |
| Minor breast surgeries for malignant neoplasms | -11’318.90 | 1’005.40 | <0.001 | -13’289.50 | -9’348.30 |
| Skin ulcer | 7’419.80 | 3’933.30 | 0.059 | -289.4 | 15’129.00 |
| Severe skin diseases | -14’058.80 | 2’635.50 | <0.001 | -19’224.40 | -8’893.10 |
| Breast plastic surgery with complex skin grafting | -6’803.50 | 2’636.40 | 0.01 | -11’970.90 | -1’636.10 |
| Infection / inflammation of the skin and subcutaneous tissue | -11’134.90 | 1’554.80 | <0.001 | -14’182.40 | -8’087.50 |
| Mild to moderate skin diseases | -11’281.40 | 2’242.70 | <0.001 | -15’677.20 | -6’885.60 |
| Skin Diseases | -15’848.00 | 2’242.50 | <0.001 | -20’243.20 | -11’452.80 |
| Adrenal surgeries for malignant neoplasms | 3’274.20 | 1’201.20 | 0.006 | 919.9 | 5’628.50 |
| Major obesity surgery with complex intervention | -1’538.70 | 1’340.60 | 0.251 | -4’166.20 | 1’088.80 |
| Thyroid, parathyroid and thyroglossal duct surgeries, without parathyroidectomies | -9’128.30 | 799.9 | <0.001 | -10’696.10 | -7’560.40 |
| Other procedures for endocrine, nutritional, and metabolic diseases | -301.1 | 1’830.40 | 0.869 | -3’888.70 | 3’286.50 |
| Surgeries on the adrenals except for malignant neoplasms and extensive lymphadenectomy | -4’143.80 | 1’307.20 | 0.002 | -6’706.00 | -1’581.60 |
| Radiation therapy for endocrine, nutritional and metabolic diseases | -5’642.40 | 6’732.80 | 0.402 | -18’838.80 | 7’553.90 |
| Complex multi-stage procedures for endocrine, nutritional, and metabolic diseases | -5’314.30 | 2’000.50 | 0.008 | -9’235.40 | -1’393.20 |
| Appendicitis unspecified | 18’139.60 | 6’731.20 | 0.007 | 4’946.40 | 31’332.90 |
| Diabetes mellitus and severe nutritional disorders | -10’780.70 | 2’479.40 | <0.001 | -15’640.30 | -5’921.10 |
| Miscellaneous Metabolic Disorders on Paraplegia/Tetraplegia | -14’286.80 | 6’730.90 | 0.034 | -27’479.30 | -1’094.20 |
| Congenital disorders of metabolism | -10’883.40 | 6’730.80 | 0.106 | -24’075.80 | 2’309.10 |
| Surgical insertion of peritoneal dialysis catheter | -11’876.10 | 1’124.10 | <0.001 | -14’079.20 | -9’672.90 |
| Kidney and ureter surgeries and major bladder surgeries for neoplasia | 6’494.20 | 1’382.80 | <0.001 | 3’783.90 | 9’204.50 |
| Kidney and ureter surgeries and major bladder surgeries except for neoplasia | -5’492.20 | 993.6 | <0.001 | -7’439.50 | -3’544.80 |
| Transurethral prostatectomy | -11’381.10 | 963.4 | <0.001 | -13’269.40 | -9’492.80 |
| Minor bladder surgery | -13’270.60 | 820.4 | <0.001 | -14’878.50 | -11’662.60 |
| Complex urethral interventions | -12’852.30 | 2’069.40 | <0.001 | -16’908.20 | -8’796.30 |
| Other surgeries for urinary tract diseases | -8’644.80 | 884.5 | <0.001 | -10’378.50 | -6’911.10 |
| Bladder reconstruction and continent pocket for neoplasm without multivisceral surgery | 23’137.20 | 1’183.90 | <0.001 | 20’816.80 | 25’457.60 |
| Complex transurethral, percutaneous-transurethral interventions | -5’229.90 | 2’149.70 | 0.015 | -9’443.40 | -1’016.40 |
| Radiotherapy for diseases and disorders of the urinary system | -14’126.50 | 6’734.90 | 0.036 | -27’327.00 | -926.1 |
| Kidney and ureter surgeries and major bladder surgeries for neoplasia | -2’143.30 | 869.6 | 0.014 | -3’847.90 | -438.8 |
| Other urethral surgeries | -14’088.20 | 998.5 | <0.001 | -16’045.20 | -12’131.10 |
| Complex transurethral, percutaneous-transurethral, and other complex retroperitoneal surgeries | -13’131.30 | 763.8 | <0.001 | -14’628.30 | -11’634.20 |
| Transurethral surgeries except prostatectomy and complex ureterorenoscopy with lithotripsy | -12’049.50 | 914.5 | <0.001 | -13’842.00 | -10’256.90 |
| Transurethral surgeries except prostatectomy and complex ureterorenoscopies | -12’628.30 | 761.9 | <0.001 | -14’121.70 | -11’134.80 |
| Complex multi-stage procedures for diseases and disorders of the urinary tract | 17’289.70 | 6’730.80 | 0.01 | 4’097.20 | 30’482.20 |
| Multivisceral intervention for diseases and disorders of the urinary organs | 22’667.40 | 2’243.10 | <0.001 | 18’271.00 | 27’063.90 |
| Complicating procedures with specific surgery or multivisceral intervention for diseases and disorders of the urinary tract | 8’600.80 | 3’427.00 | 0.012 | 1’883.90 | 15’317.70 |
| Diagnostic Ureterorenoscopy | -13’583.60 | 925.9 | <0.001 | -15’398.40 | -11’768.80 |
| Extracorporeal shock wave lithotripsy for urinary calculosis | -13’473.40 | 1’124.10 | <0.001 | -15’676.60 | -11’270.20 |
| Chronic renal disease | -3’903.80 | 1’109.70 | <0.001 | -6’078.90 | -1’728.80 |
| Neoplasms of the urinary tract | -13’753.70 | 1’219.00 | <0.001 | -16’143.00 | -11’364.70 |
| Urinary tract infections | -10’688.90 | 945.6 | <0.001 | -12’542.30 | -8’835.50 |
| Urinary calculosis and urinary tract obstruction | -13’384.90 | 775.6 | <0.001 | -14’905.10 | -11’864.60 |
| Other moderately severe diseases of the urinary system or bladder paralysis | -14’329.60 | 1’188.80 | <0.001 | -16’659.60 | -11’999.50 |
| Other serious diseases of the urinary tract | -8’123.60 | 2’149.70 | <0.001 | -12’337.00 | -3’910.20 |
| Diseases and disorders of the urinary system | -15’653.70 | 927 | <0.001 | -17’470.60 | -13’836.80 |
| Renal failure with dialysis | -16’037.20 | 3’426.40 | <0.001 | -22’753.00 | -9’321.40 |
| Major operations on the male pelvis | 4’124.20 | 864.9 | <0.001 | 2’429.10 | 5’819.40 |
| Transurethral prostatectomy | -10’971.30 | 799.6 | <0.001 | -12’538.60 | -9’404.00 |
| Penis surgery | -12’702.40 | 1’936.70 | <0.001 | -16’498.40 | -8’906.50 |
| Testicle surgery | -12’370.30 | 835.4 | <0.001 | -14’007.80 | -10’732.90 |
| Circumcision and other penile procedures | -15’617.70 | 874.7 | <0.001 | -17’332.10 | -13’903.30 |
| Other male genital procedures | -9’821.90 | 2’350.90 | <0.001 | -14’429.70 | -5’214.10 |
| Male genital procedures for malignant neoplasms | -9’304.70 | 919.3 | <0.001 | -11’106.60 | -7’502.80 |
| Radiation therapy for diseases and disorders of the male genital apparatus | 2’422.60 | 6’730.90 | 0.719 | -10’770.00 | 15’615.20 |
| Transurethral laser destruction of the prostate | -11’743.20 | 841.7 | <0.001 | -13’393.00 | -10’093.50 |
| Major bowel or bladder surgeries for diseases and disorders of the male genital system | 29’941.40 | 2’831.60 | <0.001 | 24’391.40 | 35’491.40 |
| Malignant neoplasms of the male genital system | -14’042.70 | 1’354.00 | <0.001 | -16’696.50 | -11’389.00 |
| Benign prostatic hyperplasia | -15’424.60 | 1’999.10 | <0.001 | -19’342.80 | -11’506.30 |
| Infection / inflammation of the male genital system | -12’124.20 | 2’479.40 | <0.001 | -16’983.90 | -7’264.60 |
| Other diseases of the male genital apparatus and male sterilization | -15’299.90 | 3’426.70 | <0.001 | -22’016.30 | -8’583.60 |
| Pelvic evisceration in women and radical vulvectomy or specific lymphadenectomy | 7’510.00 | 991 | <0.001 | 5’567.70 | 9’452.30 |
| Interventions on uterus and adnexa for malignant neoplasms of the ovary or adnexa | -4’154.60 | 1’017.80 | <0.001 | -6’149.40 | -2’159.80 |
| Interventions on uterus and adnexa for malignant neoplasms of other organs | -1’550.70 | 1’029.50 | 0.132 | -3’568.50 | 467.1 |
| Hysterectomy except for malignant neoplasms | -5’536.40 | 783.8 | <0.001 | -7’072.80 | -4’000.10 |
| Ovariectomy and complex tube surgeries except for malignant neoplasms | -8’413.10 | 799.3 | <0.001 | -9’979.80 | -6’846.40 |
| Complex reconstructive surgery on the female genital apparatus | -9’025.10 | 884.4 | <0.001 | -10’758.60 | -7’291.60 |
| Other uterine and adnexal surgeries except for malignant neoplasms | -8’955.50 | 915.8 | <0.001 | -10’750.40 | -7’160.60 |
| Endoscopic procedures on the female genital apparatus | -8’452.10 | 1’639.20 | <0.001 | -11’665.00 | -5’239.20 |
| Other vagina, cervix and vulva surgeries or brachytherapy for diseases and disorders of the female genital system | -12’888.50 | 918.8 | <0.001 | -14’689.40 | -11’087.60 |
| Diagnostic scraping, hysteroscopy, sterilization | -14’184.90 | 920.1 | <0.001 | -15’988.20 | -12’381.50 |
| Other procedures on the female genital apparatus with specific intervention | -1’721.70 | 876.9 | 0.05 | -3’440.50 | -3 |
| Major operations on vagina, cervix and vulva | -11’508.80 | 1’315.90 | <0.001 | -14’088.10 | -8’929.50 |
| Hysterectomy and pelvic floor plastics except for malignant neoplasms | -7’498.20 | 1’114.60 | <0.001 | -9’682.80 | -5’313.60 |
| Radiation therapy for diseases and disorders of the female genital system | -12’875.40 | 2’149.90 | <0.001 | -17’089.20 | -8’661.50 |
| Hysterectomy except for malignant neoplasms | -5’450.40 | 853.7 | <0.001 | -7’123.70 | -3’777.00 |
| Other reconstructive procedures on the female genital apparatus | -12’037.90 | 815.3 | <0.001 | -13’635.90 | -10’439.80 |
| Other uterine and adnexal surgeries except for malignant neoplasms | -10’402.20 | 846.7 | <0.001 | -12’061.80 | -8’742.60 |
| Multi-stage surgeries or complicating procedures with specific surgery for diseases and disorders of the female genital tract | 9’209.30 | 3’934.00 | 0.019 | 1’498.60 | 16’920.00 |
| Major bowel or bladder surgeries for diseases and disorders of the female genital system | 9’984.40 | 1’608.80 | <0.001 | 6’831.00 | 13’137.70 |
| Malignant neoplasms of the female genital apparatus | -11’810.30 | 1’487.50 | <0.001 | -14’725.80 | -8’894.70 |
| Infection and inflammation of the female genital tract | -14’369.80 | 2’242.60 | <0.001 | -18’765.40 | -9’974.20 |
| Menstrual disorders and other diseases of the female genital system with complex diagnostics | -14’715.20 | 1’058.50 | <0.001 | -16’789.90 | -12’640.50 |
| Caesarean section with multiple complicating diagnoses | -8’648.70 | 767.5 | <0.001 | -10’153.10 | -7’144.40 |
| Vaginal delivery with complicated procedure | -7’949.40 | 1’609.90 | <0.001 | -11’104.90 | -4’793.90 |
| Ectopic pregnancy | -8’551.00 | 1’786.20 | <0.001 | -12’051.90 | -5’050.10 |
| Hospitalization after childbirth or abortion | -13’844.80 | 1’744.70 | <0.001 | -17’264.50 | -10’425.10 |
| Cervical ostium cerclage and closure | -10’168.70 | 1’831.60 | <0.001 | -13’758.70 | -6’578.70 |
| Abortion with dilatation and curettage, by aspiration or hysterotomy | -12’857.50 | 1’254.90 | <0.001 | -15’317.00 | -10’397.80 |
| Vaginal delivery with multiple complicating diagnoses of which at least one is severe | -6’984.40 | 1’609.80 | <0.001 | -10’139.60 | -3’829.20 |
| Hospitalization after childbirth or abortion without interventional procedure | -12’560.20 | 3’083.70 | <0.001 | -18’604.20 | -6’516.10 |
| Abortion without dilation and curettage, by aspiration or hysterotomy | -8’533.30 | 6’731.30 | 0.205 | -21’726.80 | 4’660.20 |
| Other preparative hospitalization with extremely serious or severe comorbidities or complex diagnostics | -13’479.80 | 2’480.40 | <0.001 | -18’341.50 | -8’618.10 |
| Spleen surgery | -6’719.50 | 2’832.40 | 0.018 | -12’271.00 | -1’168.00 |
| Miscellaneous procedures for blood diseases | -5’926.30 | 1’327.60 | <0.001 | -8’528.40 | -3’324.20 |
| Minor interventions for blood diseases | -12’403.60 | 1’381.80 | <0.001 | -15’111.90 | -9’695.40 |
| Diseases of the reticuloendothelial and immune systems and coagulation disorders | -14’909.90 | 6’730.80 | 0.027 | -28’102.40 | -1’717.40 |
| Lymphoma and leukemia with major operative procedures | -4’845.00 | 1’058.10 | <0.001 | -6’918.80 | -2’771.20 |
| Major or complex procedures for hematologic and solid neoplasms or lymphoma and leukemia | 18’336.40 | 2’245.80 | <0.001 | 13’934.60 | 22’738.20 |
| Other hematologic and solid neoplasms with other operative procedure | -7’386.30 | 2’635.80 | 0.005 | -12’552.60 | -2’220.00 |
| Lymphoma and leukemia with specific procedure | -3’796.80 | 1’315.60 | 0.004 | -6’375.30 | -1’218.20 |
| Other hematologic and solid neoplasms with major operative procedures | -4’035.50 | 1’051.30 | <0.001 | -6’096.00 | -1’975.00 |
| Other hematologic and solid neoplasms with specific operative procedure | -8’881.40 | 2’350.70 | <0.001 | -13’488.70 | -4’274.10 |
| Other hematologic and solid neoplasms without extremely serious or severe comorbidities | -13’317.80 | 2’830.40 | <0.001 | -18’865.30 | -7’770.20 |
| Highly complex chemotherapy with surgery for hematologic and solid malignancies | 36’973.70 | 6’730.80 | <0.001 | 23’781.20 | 50’166.20 |
| Acute myeloid leukemia with intensive chemotherapy | -9’971.20 | 4’798.20 | 0.038 | -19’375.80 | -566.6 |
| Lymphoma and non-acute leukemia with septicemia or with agranulocytosis | -3’966.30 | 1’355.30 | 0.003 | -6’622.60 | -1’309.90 |
| Other hematologic and solid neoplasms with complicating diagnosis or dialysis | -8’388.80 | 1’638.40 | <0.001 | -11’600.10 | -5’177.50 |
| Other acute leukemia with highly complex chemotherapy | -3’601.60 | 6’734.50 | 0.593 | -16’801.20 | 9’598.00 |
| Hematological and solid neoplasms | -13’845.60 | 1’188.90 | <0.001 | -16’175.80 | -11’515.40 |
| HIV disease with operative procedure | 40’124.10 | 6’731.10 | <0.001 | 26’931.10 | 53’317.10 |
| Infection on HIV disease with complex diagnostics | 9’625.50 | 6’731.20 | 0.153 | -3’567.70 | 22’818.60 |
| Other disorders on HIV disease with myocardial infarction or chronic ischemic heart disease | -8’839.10 | 6’731.20 | 0.189 | -22’032.30 | 4’354.10 |
| Infectious and parasitic diseases with complex operative procedure | 572.9 | 983.5 | 0.56 | -1’354.70 | 2’500.50 |
| Complex intensive care or complex negative pressure therapy for infectious and parasitic diseases | 30’072.40 | 2’482.00 | <0.001 | 25’207.70 | 34’937.00 |
| Septicemia with complicating procedures or on organ transplant | 9’042.90 | 1’705.40 | <0.001 | 5’700.40 | 12’385.40 |
| Postoperative or post-traumatic infections with complicating procedures or complicating diagnosis | -10’870.90 | 2’149.90 | <0.001 | -15’084.60 | -6’657.10 |
| Other diseases of viral origin | -10’567.80 | 4’788.60 | 0.027 | -19’953.40 | -1’182.30 |
| Other infectious and parasite diseases | 5’490.40 | 2’830.80 | 0.052 | -57.9 | 11’038.70 |
| Other infectious and parasitic diseases | -3’346.70 | 4’789.10 | 0.485 | -12’733.30 | 6’039.90 |
| Alcohol intoxication and withdrawal or abuse-induced disorders | -7’315.00 | 6’731.20 | 0.277 | -20’508.10 | 5’878.10 |
| Polytrauma with assisted breathing or craniotomy, or complex negative pressure therapy | 26’283.60 | 1’674.50 | <0.001 | 23’001.40 | 29’565.70 |
| Polytrauma with specific interventions, complicating procedures, or interventions on multiple locations | 11’443.90 | 993.6 | <0.001 | 9’496.40 | 13’391.50 |
| Polytrauma without significant intervention | -4’539.70 | 3’428.80 | 0.186 | -11’260.20 | 2’180.80 |
| Trauma reconstruction surgeries with complicating procedures | -4’285.80 | 951.5 | <0.001 | -6’150.70 | -2’420.80 |
| Other operations for traumatisms of the lower limbs | -7’416.00 | 2’150.00 | <0.001 | -11’630.00 | -3’201.90 |
| Other interventions for traumatisms of the hand, with complex intervention | -11’280.70 | 2’069.80 | <0.001 | -15’337.60 | -7’223.90 |
| Other interventions for other traumatisms | -4’889.40 | 858.1 | <0.001 | -6’571.20 | -3’207.50 |
| Reimplantation for traumatic amputation, with reimplantation of more than one toe or hand | -12’557.00 | 6’730.80 | 0.062 | -25’749.50 | 635.4 |
| Traumatism and allergic reactions | -10’774.70 | 1’638.40 | <0.001 | -13’986.00 | -7’563.50 |
| Poisonings and toxic effects of drugs, medications and other substances or consequences of medical treatment | -11’481.40 | 1’252.90 | <0.001 | -13’937.20 | -9’025.70 |
| Other disease caused by trauma, poisoning or toxic effect | -14’690.30 | 2’635.90 | <0.001 | -19’856.80 | -9’523.90 |
| Surgery or assisted breathing | -9’659.80 | 6’731.10 | 0.151 | -22’852.80 | 3’533.30 |
| Other burns with skin graft on septicemia or with complicating procedures | -3’230.40 | 1’999.20 | 0.106 | -7’148.90 | 688 |
| Other burns with other surgeries | -2’321.30 | 2’636.30 | 0.379 | -7’488.40 | 2’845.80 |
| Other burns | -4’954.50 | 2’070.10 | 0.017 | -9’011.90 | -897.1 |
| Operative procedures for other conditions requiring the use of health care services | -8’671.70 | 1’743.20 | <0.001 | -12’088.50 | -5’254.90 |
| Other factors affecting health status and further treatment after completion of treatment | -15’497.30 | 2’479.20 | <0.001 | -20’356.50 | -10’638.00 |
| Disorders, symptoms, other abnormalities, and further treatment with specific diagnosis and procedure or dialysis | 6’090.90 | 3’933.90 | 0.122 | -1’619.60 | 13’801.40 |
